# A diagonal volatility basis set to assess the condensation of organic vapors onto particles†

**DOI:** 10.1039/d5ea00062a

**Published:** 2025-07-21

**Authors:** Brandon Lopez, Nirvan Bhattacharyya, Jenna DeVivo, Mingyi Wang, Lucia Caudillo-Plath, Mihnea Surdu, Federico Bianchi, Zoé Brasseur, Angela Buchholz, Dexian Chen, Jonathan Duplissy, Xu-Cheng He, Victoria Hofbauer, Naser Mahfouz, Vladimir Makhmutov, Ruby Marten, Bernhard Mentler, Maxim Philippov, Meredith Schervish, Dongyu S. Wang, Stefan K. Weber, André Welti, Imad El Haddad, Katrianne Lehtipalo, Markku Kulmala, Douglas Worsnop, Jasper Kirkby, Roy L. Mauldin, Dominik Stolzenburg, Siegfried Schobesberger, Richard Flagan, Neil M. Donahue

**Affiliations:** a Carnegie Mellon University Department of Chemistry Pittsburgh PA USA nmd@andrew.cmu.edu +1 412 268-4415; b University of Chicago Department of the Geophysical Sciences Chicago IL USA; c Institute for Atmospheric and Environmental Sciences, Goethe University Frankfurt 60438 Frankfurt am Main Germany; d Center for Energy and Environmental Sciences, Paul Scherrer Institute 5232 Villigen Switzerland; e Institute for Atmospheric and Earth System Research/Physics, University of Helsinki Helsinki 00014 Finland; f Department of Technical Physics, University of Eastern Finland PO Box 1627 70211 Kuopio Finland; g Yusuf Hamied Department of Chemistry, University of Cambridge Cambridge UK; h Lebedev Physical Institute of the Russian Academy of Sciences 119991 Moscow Russia; i Moscow Institute of Physics and Technology (National Research University) 141701 Moscow Russian Federation; j Ion Molecule Reactions & Environmental Physics Group Institute of Ion Physics and Applied Physics Leopold-Franzens University Innsbruck Technikerstraße 25 A-6020 Innsbruck Austria; k CERN, The European Organization for Nuclear Research Geneve 23 CH-1211 Switzerland; l Finnish Meteorological Institute Helsinki Finland; m Aerodyne Inc Billerica MA USA; n Department of Chemistry, CIRES, University of Colorado Boulder Boulder CO 80309-0215 USA; o Faculty of Physics, University of Vienna 1090 Vienna Austria; p Institute of Materials Chemistry TU Wien 1060 Vienna Austria; q Division of Chemistry and Chemical Engineering, California Institute of Technology Pasadena CA 91125 USA; r School of Atmospheric Sciences, Sun Yat-sen University Zhuhai China

## Abstract

We present a “diagonal” Volatility Basis Set (dVBS) comparing gas-phase concentrations of oxygenated organic molecules (OOM) to their condensed-phase mass fractions. This permits closure of vapor concentrations with particle composition constrained by particle growth rates, allowing the contributions of quasi non-volatile condensation, equilibrium partitioning, and reactive uptake to be separated. The dVBS accommodates both equilibrium and dynamical (growth) conditions. Growth implies an association between gas and particle concentrations governed by a “condensation line” that is set by the particle growth rate, which fixes the total (excess) concentration of condensible vapors. The condensation line defines an infeasible region of high particle mass fraction and low gas concentration; under steady-state growth conditions, compounds cannot appear in this infeasible region without being formed by condensed-phase chemistry. We test the dVBS with observations from the CLOUD experiment at CERN using data from a FIGAERO I^−^ Chemical Ionization Mass Spectrometer measuring vapors directly and particle composition *via* temperature programmed desorption from a filter. A dVBS analysis finds that data from an α-pinene + O_3_ run at 243 K are consistent with volatility driven condensation forming the large majority of particle mass, with no compounds clearly within the infeasible region.

## Abstract 2

We present a “diagonal” Volatility Basis Set (dVBS) comparing gas-phase concentrations of oxygenated organic molecules (OOM) to their condensed-phase mass fractions.

## Graphical abstract

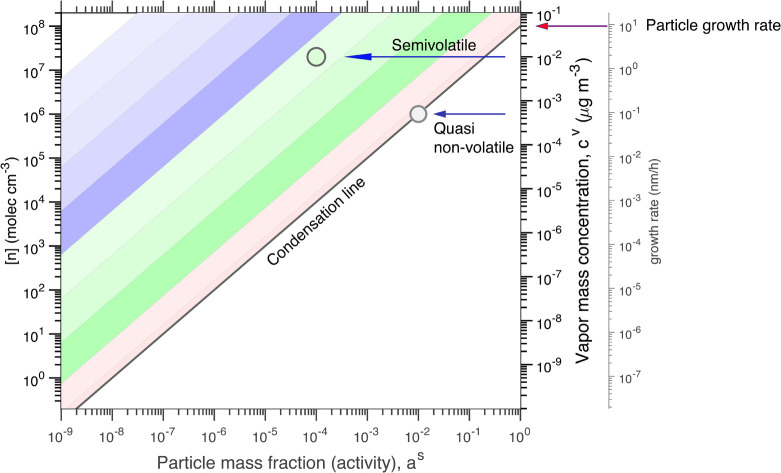

**Environmental significance**

Condensation of vapors drives particle growth and much of the total particle mass in the atmosphere. For organics, this can involve thousands of molecules, and closure of what species are driving growth remains elusive. This in turn means we do not yet know what precursors are responsible for the growth, nor whether it is primarily governed by gas-phase chemistry preceding the condensation or condensed-phase chemistry following the condensation. Using the method presented here, researchers will be able to test experiments on realistic, complex systems for closure identifying the key processes governing particle growth.

## 1 Introduction

Particle growth rates are fundamental to understanding atmospheric aerosols. The survival probability of nanoparticles depends exponentially on the ratio of growth rate to the condensation sink (the collision frequency of nanoparticles with all particle surface area, technically the coagulation sink).^1,2^ Because of this, growth rates are just as important as nucleation rates when it comes to understanding the production rate and thus overall number concentration of cloud condensation nuclei (CCN).^3–5^ Even many primary emissions consist of particles well below CCN size. Finally, condensing molecules are often highly soluble, enhancing cloud droplet activation,^6^ so condensational growth underlies almost all CCN.^7,8^

Organic compounds are often responsible for most particle growth, especially in the continental boundary layer.^9–11^ Organics comprise roughly half of the particle mass throughout much of the remote atmosphere, with sulfate making up much of the rest.^12,13^ Sulfate mass arises from H_2_SO_4_ condensation as well as aqueous-phase SO_2_ oxidation, whereas the organic mass arises from a vast array of condensing vapors.^14^ There are important open questions concerning the processes that govern this growth. What fraction of particle growth driven by organic condensation is rate-limited by the collision frequency with particles (*i.e.* is effectively irreversible), what fraction is rate-limited by the volatility of the condensing organics, and what fraction is rate-limited by subsequent reactions in the condensed phase?^15^

Here we shall consider these questions and develop diagnostics, emphasizing simultaneous measurements of gas-phase concentrations (activities) and condensed-phase composition (also activities) under conditions where the particle growth rates are also well constrained. Our goal is to build on the Volatility Basis Set (VBS),^16–19^ extending it to the dynamical VBS.^20–22^ We give explicit consideration to steady-state conditions during particle growth in contrast to equilibrium conditions. We shall present extensive thermodynamics and microphysics in order to build a representation of coupled gas- and particle-phase composition, along with growth rates, that can identify key observables that could identify (or rule out) various processes associated with particle growth, for example simple condensation, delayed uptake, reversible condensed-phase chemistry, and irreversible condensed-phase chemistry.

## 2 Notation

Here we shall refer to concentrations, *c*, of a species, *i*, either in the vapor phase, v, or a suspended particle phase, s, where the specific particle population, *p*, has properties such as diameter, *d*_*p*_, total number, *N*_*p*_, as well as a composition, total mass, *etc.* We will designate the relevant phases with a superscript and the specific entities (the species or particle population) with subscripts. For example, *c*^s^_*i*,*p*_ means “the concentration of species *i* in (suspended) particle population *p*”, and *ϕ*^v,s^_*i*,*p*_ means “the flux (per unit particle surface area) from the vapor to the suspended particle phase of species *i* in particle population *p*”. When subscripts are dropped this indicates summation over all entities, so *ϕ*^v,s^_*p*_ means “the flux of all species (per unit particle surface area) from the vapors to suspended particle population *p*”, and *Φ*^v,s^_*i*_ means “the total net flux of species *i* to all particles”. We shall also use superscripts to identify properties (*i.e. R*^gr^ for growth rate, *k*^I^ for first-order coefficient, *etc.*). A full description is in the Abbreviations section.

## 3 Microphysics

Particle microphysics is often developed for larger particles in the continuum fluid regime (with Knudsen number *K*_n_ ≪ 1) and then corrected for non-continuum effects for small particles (*d*_*p*_ ≲ 500 nm) that fall in transition regime and ultimately the kinetic regime for the smallest particles.^20,22–24^ However, we find it convenient to develop the dynamics in the kinetic (collision-limited) regime and then correct for emerging diffusion limitations as particles grow toward the continuum regime.^21,25^ This is because the physics then emerges largely as rate limitations (*i.e.* diffusion limitations) rather than apparent enhancements, but also because it takes days for particles to nucleate and grow to 500 nm, and the large majority never reach that size.^7,8^ Most particles thus never even reach the transition regime, much less the continuum regime. These treatments are equivalent, but they do encourage different perspectives. For example, in this kinetic-regime based frame of reference, the diffusion constants of vapors are almost irrelevant, emerging only in the transition regime correction before they ultimately govern transport in the continuum regime; instead, it is vapor mass and velocity, and even relative vapor-particle reduced mass and collision speed, that governs collisions.

At its simplest, the gross flux per unit area of a species, *i*, to a suspended condensed phase, s, is given by the speed of that vapor normal to the surface, *s*^⊥^_*i*_, along with the vapor, v, with concentration *c*^v^_*i*_. At this point we do not designate a particle population, *p*, because we have yet to define its properties, and it is nominally flat with infinite mass; however, when the specific particle matters, the subscript will become *i*, *p*. Here it is a uni-directional condensation flux, →.

1 *ϕ*^v,s^_*i*,→_ = *s*^⊥^_*i*_*c*^v^_*i*_

However, the net flux to that condensed phase will be some fraction of that gross flux, given by an uptake coefficient, 0 ≤ *γ*_*i*_ ≤ 1.

2 *ϕ*^v,s^_*i*_ = *γ*_*i*_*s*^⊥^_*i*_*c*^v^_*i*_

That net flux will in turn cause the interface between the two phases, given by the height of the (suspended) surface, *z*^s^, to grow at a rate (speed, *R*^gr^_*i*_, due to *i*) given by the net flux and the molar or specific volume of the species, *v*_*i*_ (with the appropriate units, in whatever constitutes the condensed phase, with a mass or number *m*^s^_*i*_).3
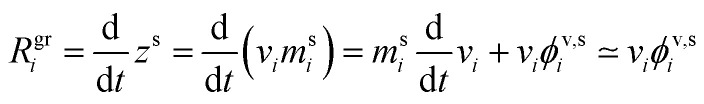


As a simplification, we assume ideal mixing and that the specific volume remains constant. The total growth rate is simply the sum over all species.4
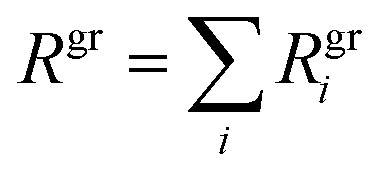


The natural frame of reference for kinetic collisions is the center of mass, with a reduced mass and a single collision parameter; however, the natural frame of reference for aerosol microphysics is the particle itself, with an effective (physical and spherical) diameter, *d*_*p*_. Microphysical expressions now refer to a species within a specific population and so are designated with a dual subscript, *i*, *p*. This causes certain terms to emerge as corrections, especially at very small particles sizes, but does not otherwise greatly influence this discussion.^21^ The (diameter) growth rate of the particles (note *d*_*p*_ = 2*r*_*p*_) is5
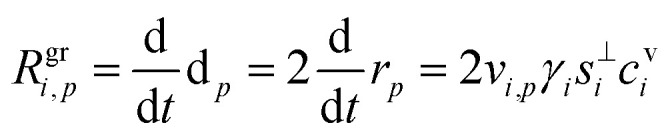


We can represent the growth as if it were driven by a single effective species to a generic surface, (*i*, *p*) → *e*, with *γ*_*e*_ = 1

6 *R*^gr^_*e*_ = *v*_*e*_*s*^⊥^_*e*_*c*^v^_*e*_

The effective perpendicular speed is known from kinetic theory.7
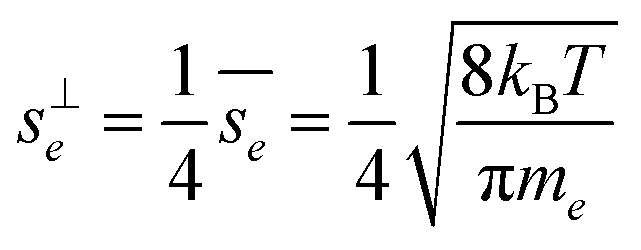


Thus, if the growth rate is known, the effective total concentration of condensing vapors is then known as well.8
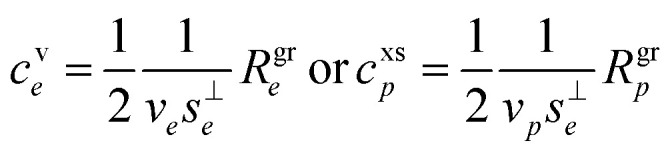


It is not necessary to reduce the growth to a single condensing species, but this provides an important limiting case throughout this analysis. It is always possible to consider the pure limit (*w*_*i*,*p*_ → *w*_*e*_ → 1), and when the particle population is known (*e* → *p*) we adopt the second form, referring to the “excess” concentration, xs, as shown, though an overall effective speed (considering the average mass of condensing vapors) still needs to be found. This is the anchor point for the “diagonal” of the dVBS distribution – tying unit condensed mass fraction to the total concentration of condensable vapors. There is very little wiggle room here; if particles are growing, molecules are condensing to them, and that flux defines the growth rate. Aside from large changes in the specific volume, nothing else can drive this growth. Even if the specific volume does change, in most cases that will be due to a condensing species (*e.g.* water). The gas-phase concentration of condensing species is unambiguously related to the growth rate.

### 3.1 Particle dynamics

Particles have some properties that affect condensation dynamics. They have a diameter, *d*_*p*_, but also a finite mass, *m*_*p*_. Likewise, condensing molecules have a finite effective diameter, *d*_*i*_, and a specific volume within the particle, *v*_*i*,*p*_. Collisions between vapors and (suspended) particles likely involve some (van der Waals) attractive interaction potential, modifying a hard-spheres collision cross section, *σ*^hs^_*i*,*p*_, by an enhancement factor, *E*^μ^_*i*,*p*_. The line-of-centers collision speed, *s̄*_*i*,*p*_, differs from the molecular speed, *s̄*_*i*_, by a factor, *e*_*i*,*p*_, derived from the reduced mass, and the finite size of the molecule also contributes to the hard-spheres impact parameter and enhances collisions by a factor, *ε*_*i*,*p*_. Some collisions may also bounce, resulting in a non-unit mass accommodation coefficient, *α*_*i*,*p*_ < 1, and for larger particles there may be a diffusion limitation, *B*_*i*,*p*_, in the gas-phase layer surrounding the particle.^21^

Importantly, condensing (or evaporating) species have a volatility, given by a saturation concentration, 
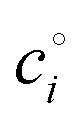
. This saturation concentration can also be modified by particle curvature, leading to a Kelvin term that can be expressed in terms of a decadal Kelvin diameter (the diameter at which the pure saturation vapor pressure is one order of magnitude higher than over a flat surface).9
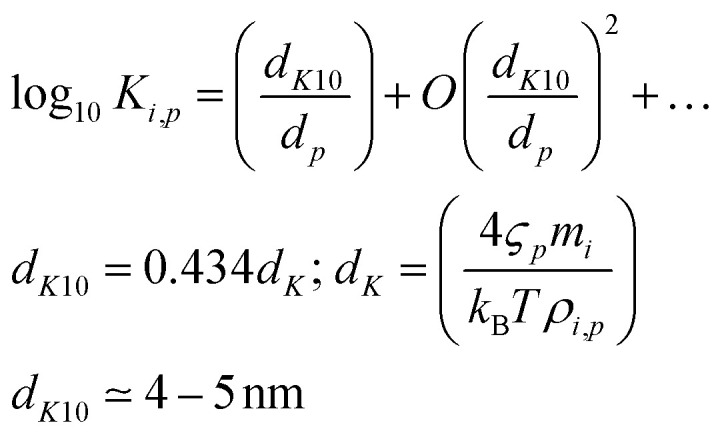


Formally the Kelvin term is part of the (suspended) condensed-phase activity, *a*^s^_*i*,*p*_, but we separate it explicitly to emphasize its role in – very small – particles. With typical Kelvin diameters of order 5 nm, the Kelvin effect is only important for very small particles (smaller than 10 nm or so). This is because organic vapors have volatilities spanning many orders of magnitude and thus the Kelvin term is only important when it is greater than 10 or so. This only occurs for very small particles; however, then it is extremely important.^20,22,26^

The condensed-phase activity can be defined with respect to either the condensed-phase mole fraction, *x*_*i*,*p*_, or the condensed-phase mass fraction, *w*_*i*,*p*_; either is multiplied by the appropriate activity coefficient, *ζ*_*i*,*p*_; here we use *ζ* instead of the conventional *γ* to avoid confusion with the uptake coefficient.^16^ Regardless, provided that the fraction is defined with respect to the measured concentration units:10
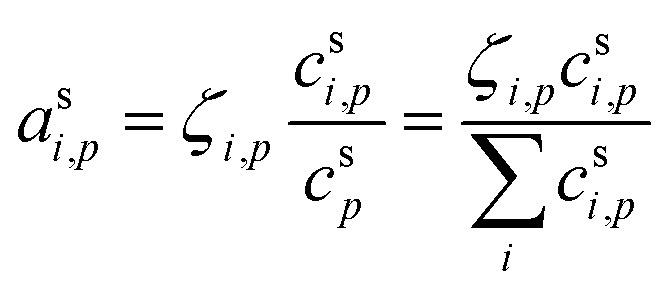


The mass (or mole) balance constraint is that the sum of the relevant condensed-phase fractions for all constituents must be unity: 
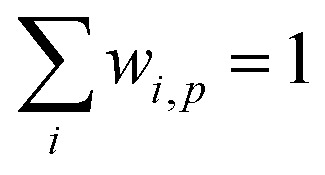
.

With those terms in mind, the net condensation flux to a suspension of identical particles with a known number concentration, *N*^s^_*p*_, can be written in several ways, starting with the fundamental equation base on collisions between particles and vapors, but ending with deposition of vapors to the particle surface area. These are all equivalent, but each can be useful in different contexts.11
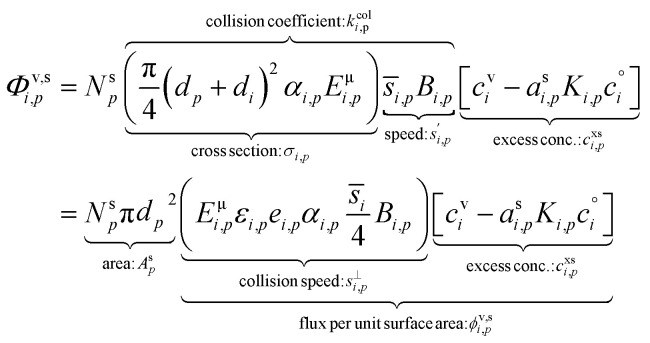


For particle growth, the important context is the flux of that species per unit particle surface area. This in turn can be affected by the particle-phase activity and thus leads to the uptake coefficient.12
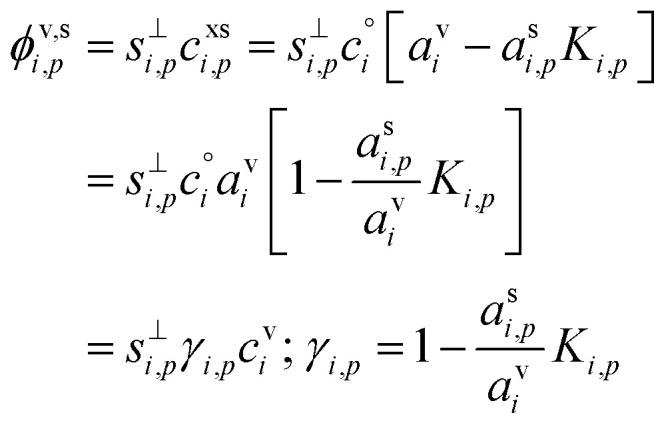


The uptake coefficient, *γ*_*i*,*p*_, is the fractional excess saturation ratio over a small, curved particle: the ratio of the “Kelvin adjusted” suspended-phase activity, *a*^s^_*i*_*K*_*i*,*p*_, to the equilibrium suspended-phase activity, *a*^s,eq^_*i*,*p*_ = *a*^v^_*i*_. The excess activity is conceptually the same as the excess concentration; it is the amount of material in the vapor phase in excess of equilibrium at any given time. If the system were at equilibrium, no collisions would result in uptake (*γ*^cond^_*i*,*p*_ = 0) whereas for kinetic uptake every collision would be taken up (*γ*^cond^_*i*,*p*_ = 1). In general we can write13
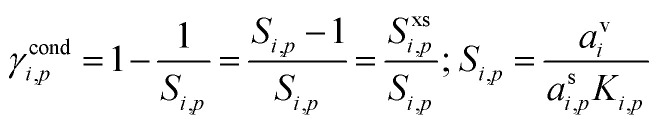
*S*_*i*,*p*_ is the saturation ratio of the vapor to the suspended particle phase (including curvature), and the excess saturation ratio is *S*^xs^_*i*,*p*_ = *S*_*i*,*p*_ − 1. The (condensation) uptake coefficient is thus just the fractional excess saturation ratio.

Net condensation is actually controlled by the surface activity, *a*^s,u^_*i*,*p*_, which may differ from the bulk activity, *a*^s,b^_*i*,*p*_, especially when diffusion within the particles is slow.^27–32^ This can be very important for semi-volatile species (*S*^xs^_*i*,*p*_ ≤ 1), especially for reactive uptake.^27,28,33^ However, when a particle (and thus the interface) is growing, and when the vapors have high saturation ratios (*S*^xs^_*i*,*p*_ ≫ 1), the particle activity is irrelevant. We consider both cases below, with any condensed-phase processes influencing the overall uptake coefficient (0 ≤ *γ*_*i*,*p*_ ≤ 1). This also applies to phase-separated particles, where at equilibrium the activities in each phase must be equal (*a*^s,1^_*i*,*p*_ = *a*^s,2^_*i*,*p*_ ≤ 1).

Various sums (or integrals over distributions) determine: the total flux of all species to these suspended particles per unit surface area, *ϕ*^v,s^_*p*_, or to the total population, *Φ*^v,s^_*p*_; the flux of just the one species to the entire suspended ensemble of particles, *Φ*^v,s^_*i*_; and finally the flux of all species to all particles, *Φ*^v,s^:14
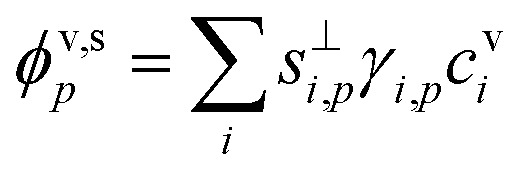

15 *Φ*^v,s^_*p*_ = *N*^s^_*p*_π*d*_*p*_^2^*ϕ*^v,s^_*p*_

16
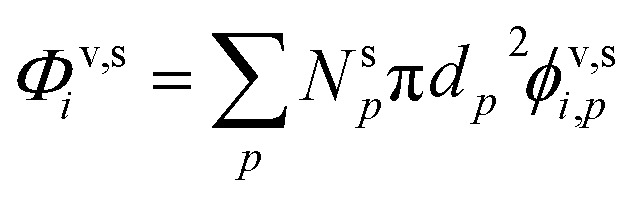
17
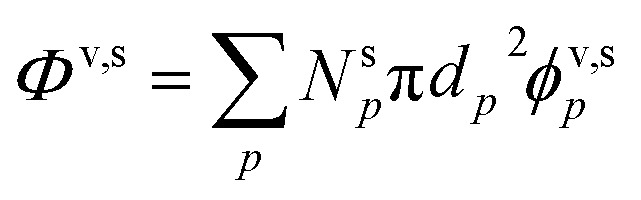
The net flux is governed by an excess (vapor) concentration, *c*^xs^_*i*,*p*_, which we can express as the vapor concentration and a condensation uptake coefficient, *γ*^cond^_*i*,*p*_. The uptake coefficient can also be used to define an (apparent) uptake speed (*s*^up^_*i*,*p*_ = *s*^⊥^_*i*_*γ*^cond^_*i*,*p*_). Because our context is to understand growth rates, these derivations focus on speed, with various effects lowering the maximum speed from the line-of-centers collision speed of vapors with particles, but an anchor point being the average speed of a vapor in the gas phase; various influences can lower this effective speed and thus the growth rate, but it fundamentally anchors the problem.

The condensation sink is an important parameter in many contexts; it is the collision frequency of vapors with the full particle distribution, including the mass accommodation coefficient, *α*_*i*,*p*_. It governs the vapor concentration and the maximum timescale for vapors to approach steady state. Highly volatile species can equilibrate faster, but the condensation sink gives the maximum timescale.^34,35^ The condensation sink is a first-order loss coefficient for vapors (a frequency) and so we use a rate-coefficient symbol, *k*, for consistency.18
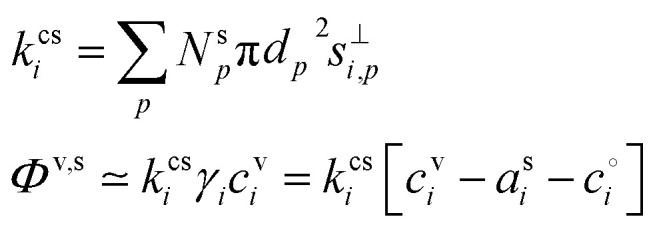
In eqn (18) we can use the condensation sink to find the total flux of a given vapor to particles, provided the particle composition effects are uniform. The condensation sink is also often expressed as the Fuchs corrected surface area multiplied by an average speed, if the transition-regime correction factor, *B*_*i*,*p*_, is applied to the surface area rather than the collision speed as shown in eqn (11).^24^ Though it is always important for a full understanding of the coupled system, in this context of understanding growth rates, the condensation sink does not directly influence individual particles and does not need to be known if vapors are directly measured.

#### 3.1.1 Growth rate

The contribution of a species, *i*, to the growth rate, *R*^gr^_*i*,*p*_, is proportional to the flux, *ϕ*^v,s^_*i*,*p*_. Assuming a spherical particle (or a spherical equivalent *d*_p_), and using the molar or specific volume appropriate to the concentration units, we find (for constant specific volume)19
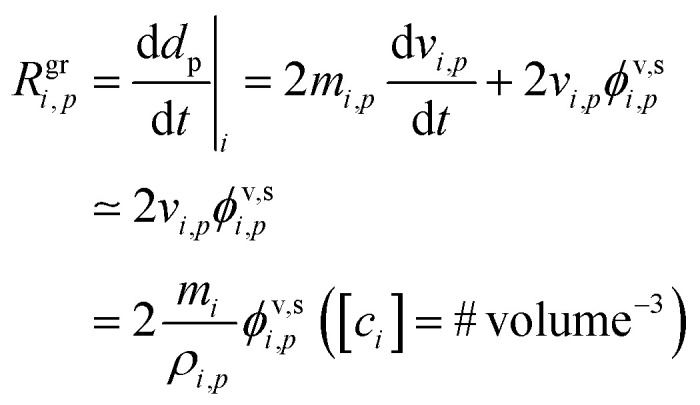
20
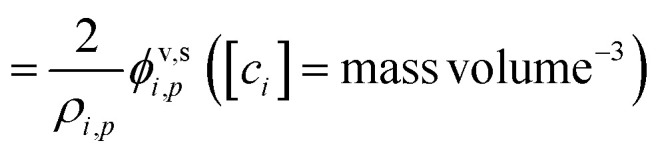


Mass concentration units (typically μg m^−3^) are common and in many ways more natural for this application, and so we shall use them as our primary concentration measurement. This is both because the molar mass, *m*_*i*_, is not used in the growth rate above and also because the mass-based activity is the same as the volume fraction for a constant density, *ρ*_*i*,*p*_.21
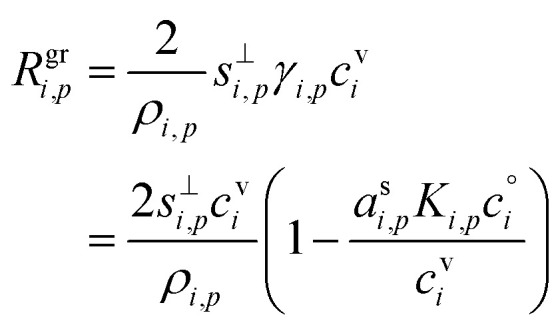
22
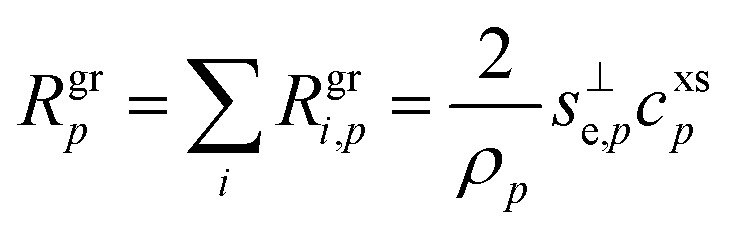
23
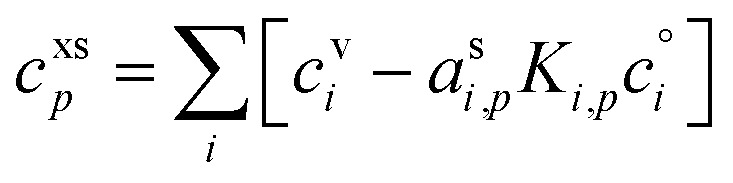
As discussed for eqn (8), the total excess vapor concentration, *c*^xs^_*p*_ is the effective concentration, *c*^v^_*e*_, of a species that would condense irreversibly (*γ* = 1) to drive a given growth rate. The collision speed, *s*^⊥^_*i*,*p*_, is derived from the average molecular speed, and at the kinetic limit (10 ≲ *d*_p_ ≲ 30 nm), *s*^⊥^_*i*,*p*_ ≃ *s̄*_*i*_/4.

The effective condensible vapor concentration is a critically important quantity. Typical observed particle growth rates range between 1–100 nm h^−1^ but are often 10 nm h^−1^ or less.^10,11^ For condensation of Low Volatility Organic Carbon (LVOC) vapors, we can consider typical values to be *ρ*_*p*_ ≃ 1.4 g cm^−3^ = 1.4 × 10^12^ μg m^−3^ and a molar mass of *m*_*i*_ ≃ 0.3 kg mole^−1^ = 5 × 10^−25^ kg. This gives a mean speed of *s̄*_*i*_/4 = 36 ms^−1^ and

24 *R*^gr^_*p*_ = 5 × 10^−11^ (ms^−1^ μg^−1^ m^3^)*c*^v^_*e*_ = 184(nm h^−1^ μg^−1^ m^3^)*c*^v^_*e*_

Thus, for *R*^gr^_*p*_ = 10 nm h^−1^,

25 *c*^v^_*e*_ = 0.05 μg m^−3^

If these condensible vapors have 
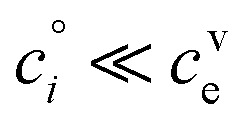
 then *a*^v^_*i*_ ≫ 1 and *γ*_*i*_ ≃ 1.

## 4 Dynamics

In any context, the vapor and suspended particle concentrations of each species evolve through coupled differential equations.26
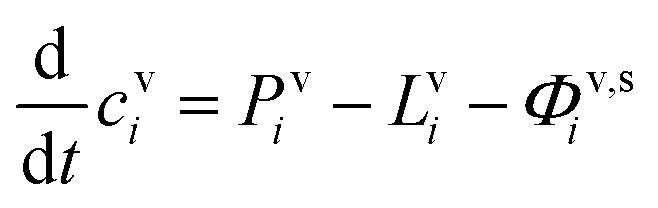
27
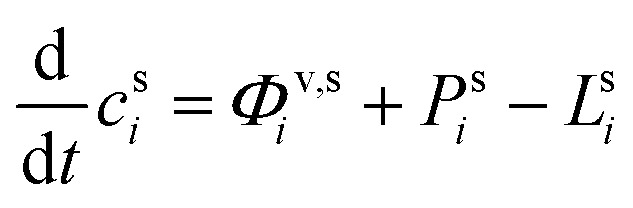


The flux is nominally from the vapor to the suspended particle phase and so appears as a sink in the vapor eqn (26) and a source in the particle eqn (27). This is because condensational growth predominates in the atmosphere; however, net condensation or net evaporation is possible, and not every species must have net flows in the same direction. Specifically, if non-reactive condensation is the only process affecting the particles, then *P*^s^_*i*_ = *L*^s^_*i*_ = 0, and if, further, the vapor concentrations are constrained by observation, *c*^v^_*i*_ = *c*^v,obs^_*i*_, then the particle behavior can be separated from the vapors without consideration of vapor production and loss. However, if there is loss in the particles, then there must be formation of at least one reaction product in the particles; this will in turn drive at least some evaporation from the particles and serve as an additional vapor source.

## 5 Thermodynamics

The formation of vapors driving particle growth is intrinsically out of equilibrium, but again in any context the system will always be evolving toward an equilibrium defined by the underlying thermodynamics.

### 5.1 Equilibrium

Equilibrium for the aerosol suspension requires no net fluxes (production, loss, or growth) and equal activity in all phases. Consequently, *γ*_*i*,*p*_ = 0, and from eqn (12) we have28
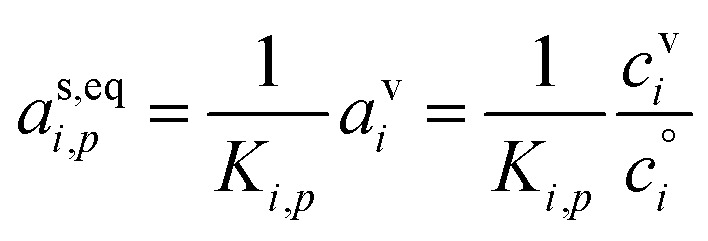
A (generally very unstable) equilibrium is possible for very small particles with *K*_*i*,*p*_ > 1, but the most important conclusion is that at equilibrium it is impossible to find a large excess of vapors because the particle-phase activity cannot exceed 1; thus 
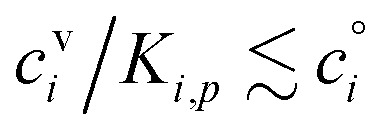
 because *a*^s^_*i*,*p*_ ≤ 1. Non-trivial vapor concentrations of very low volatility compounds are intrinsically out of equilibrium and thus imply net condensation and growth. Equally, the presence of very low volatility compounds in the vapor phase implies a source, possibly from transport (a flow in or temperature change) but more likely, and more dramatically, from chemistry.

**Fig. 1:**
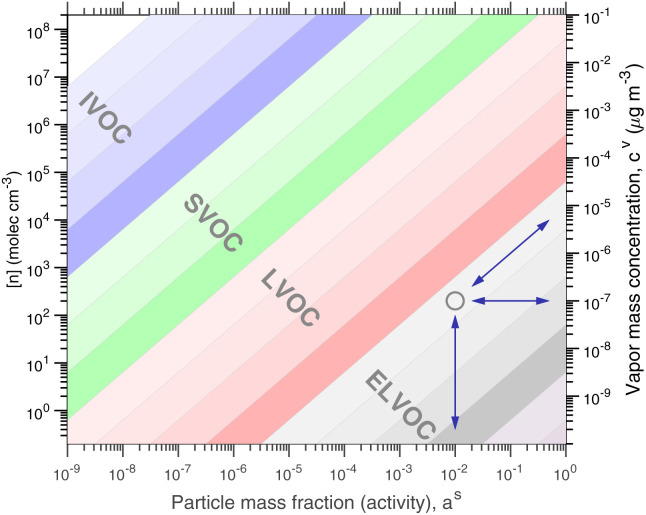
Equilibrium diagonal Volatility Basis Set (dVBS). The phase diagram shows vapor mass concentration, *c*^v^_*i*_, *vs.* condensed (suspended) phase mass fraction (activity), *a*^s^_*i*_. Broad volatility classes are indicated by colored bands and decadal bins shown with hue saturation, described in the text. Saturation concentrations for each bin (the center of the colored band) are found on the right-hand *y*-axis for a pure condensed phase *a*^s^_*i*_ = 1. Vapor activity and condensed-phase (suspended) activity must be equal, resulting in extremely low vapor concentrations of extremely low volatility species; an example is an ELVOC with saturation concentration *c*° = 10^−5^ μg m^−3^, condensed phase mass fraction *a*^s^ = 0.01 and equilibrium vapor mass concentration *c*^v^ = 10^−7^ μg m^−3^. The vapor is plotted with a circle, filled with a color indicating the saturation concentration; because it is at equilibrium, the color matches the color of the diagonal band corresponding to the volatility. At equilibrium, any two of these values determines the third.

Fig. 1 shows the equilibrium relationship between condensed-phase activity and gas-phase (vapor) concentration over a wide range of the Volatility Basis Set.^16,17,36^ This is the same relation that underlies the “classic” one-dimensional VBS,^16,37^ but focused on particle composition, *a*^s^_*i*_, rather than total suspended particle mass (*c*_OA_ = *c*^s^). The volatility bins appear as diagonal stripes with volatility classes^36,38^ indicated by various hues. The hues also show the volatility of individual species, plotted with filled circles (here a ULVOC in gray). The classes correspond to qualitative phase partitioning behavior:

**VOC**: Volatile Organic Compounds (log_10_ *c*° ≥ 6.5) are highly volatile and predominate gas-phase chemistry.

**IVOC**: Intermediate Volatility Organic Compounds (2.5 ≤ log_10_ *c*° < 6.5, blue hues) do not contribute substantially to particle mass.

**SVOC**: Semi Volatile Organic Compounds (−0.5 ≤ log_10_ *c*° < 2.5, green hues) equilibrate with significant mass in both phases.

**LVOC**: Low Volatility Organic Compounds (−4.5 ≤ log_10_ *c*° < −0.5, salmon hues) are mostly in the particle phase at equilibrium, but their volatility matters.

**ELVOC**: Extremely Low Volatility Organic Compounds (−8.5 ≤ log_10_ *c*° < −4.5, gray hues) are almost exclusively in the particle phase at equilibrium, but they do not nucleate.

**ULVOC**: Ultra Low Volatility Organic Compounds (log_10_ *c*° < −8.5, plum hues) cluster and nucleate.

The diagonal stripes provide the name for the diagonal Volatility Basis Set, “dVBS”, which as we shall see is always modified by the dynamical growth conditions – here “equilibrium” for zero growth. The Raoult's law mixing behavior of each forms a diagonal band in this log–log plot, with the saturation concentration 
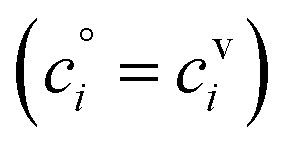
 for a pure compound (on the right-hand limit) when *a*^s^_*i*_ = 1. For reference the figure locates an ELVOC with 
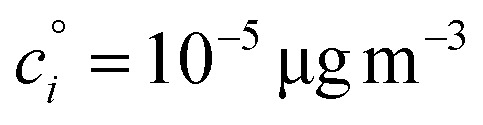
 and *a*^s^_*i*_ = 0.01; any two values of the vapor concentration (right *y*-axis), particle activity (mass fraction, *x*-axis) and saturation concentration (*y*-value extended diagonally to the right-hand limit) constrain the third, as indicated by the blue arrows.

In the dVBS, the right-hand *y*-axis at *a*^s^ = 1 is primary, and the left-hand (number concentration) axis is only representative for a typical molar mass (here 250 amu). This continues throughout this discussion; we present dVBS with multiple *y* axes, where any (or all) could be exactly known; however, symbols are only plotted with reference to one primary axis, with the others providing approximate values for reference. This could also apply to the *x*-axis, with activity, *a*^s^_*i*,*p*_, and mass fraction, *w*_*i*,*p*_, but in this discussion we only present assumed ideal solutions.

#### 5.1.1 Temperature dependence

As shown by Epstein^39^ and discussed in Stolzenburg,^22^ the dependence of the saturation concentration on temperature can be described with an equation approximating the Clausius Clapeyron equation29
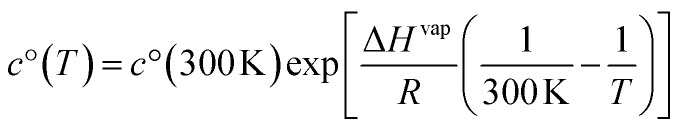
The enthalpy of vaporization is a function of “intrinsic” volatility, *c*°(300 K). It is fundamental that the vaporization enthalpy drives most volatility differences (we expect the vaporization entropy not to be dramatically or systematically variable), and an empirical relation with 
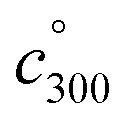
 is30
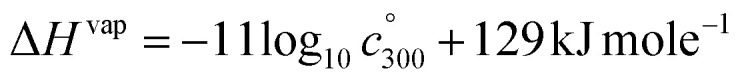
There are some theoretical reasons to favor a less severe temperature dependence with 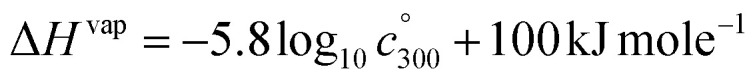.^16^ For the most part, in this theoretical discussion we simply assume a known saturation concentration at any given temperature, *T*; however, when comparing with observations the accuracy of temperature corrections will be important, especially for low-temperature conditions typical of the free troposphere.

#### 5.1.2 Equilibration timescales

It is important to consider how long it will take a system to relax to equilibrium (or to a steady state). The overall condensed-phase activity for a particle distribution from eqn (10) is31
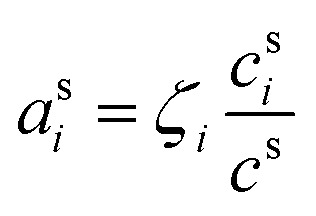
where *c*^s^ is the total mass of the phase containing organics (often written *c*_OA_, though it can contain inorganic species, notably water). If we split the flux balance into forward and reverse terms for the equilibrium case, subsume any activity coefficient into an effective saturation concentration 
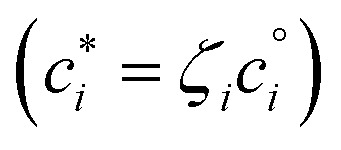
, and use eqn (18) to relate the total flux to the condensation sink, we find32
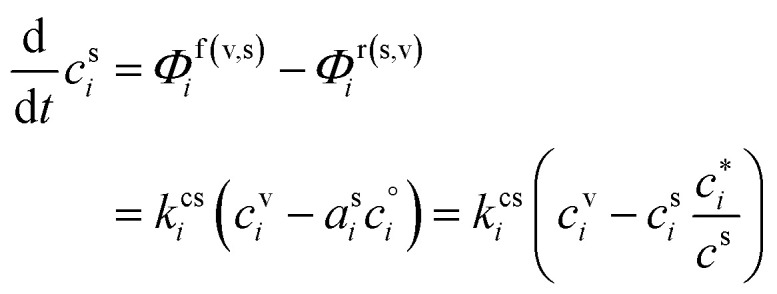
33
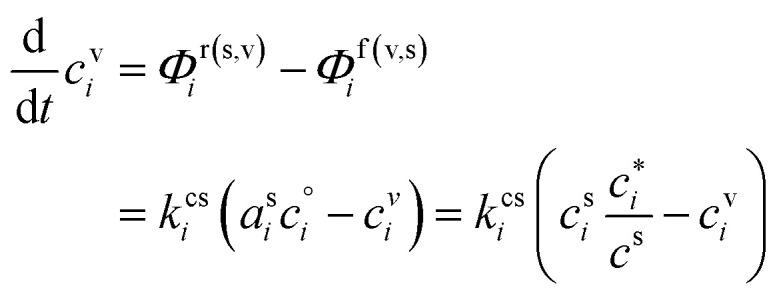


This is a classic system of the form 
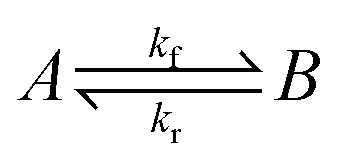
, where the eigenvalue for equilibration is34
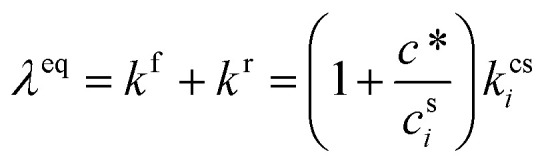


This means that the condensation sink sets a maximum timescale for equilibration ranging from 1 s^−1^ in polluted regions to 10^−3^ s^−1^ in the remote continental boundary layer and the free troposphere.^7,40^ However, (comparatively) volatile species (defined by a large ratio c*/*c*^s^_*i*_) can equilibrate much more quickly; an example is water droplets and vapor passing over an airfoil. Equilibration only occurs when the flux between the phases, *Φ*^v,s^_*i*,*p*_, is the dominant term in the differential equations for all (significant) vapors and particles. Otherwise a steady state will instead apply. A common and important example is chamber experiments with a relatively high wall collision frequency (wall loss) and a relatively low suspended condensation sink.

## 6 Steady-state growth

Systems with condensational growth are inherently out of equilibrium, but if the vapor concentrations are held constant (for example by steady flows or a steady chemical production with constant molar yields of a variety of species), then we can expect the (suspended) condensed phase composition (activities) to remain reasonably constant as well, so the system will be in steady state. Two exceptions to this are changes as the Kelvin term diminishes with growth of very small particles and slow condensed-phase chemical reactions. Although in those cases we do not expect the composition to remain constant, the constant composition case remains informative. Moreover, we could, in principle, account for changes of the Kelvin term by integrating over the entire period of particle growth. Here our objective is to develop diagnostics to present simultaneous vapor and particle-phase observations that we can relate to the equilibrium expectations of Fig. 1. Consequently, we develop formal expressions for the steady-state particle composition (and activities), given constrained vapor concentrations of all condensing species. We then explore several cases isolating the signatures of different rate-limiting phenomena.

The quasi-steady state composition of growing particles is given by a steady-state activity, *a*^s,ss^_*i*,*p*_, for a given fixed mixture of vapors, {*c*^v^_*i*_}. The activity coefficients will not change with steady composition, so the steady-state condensed-phase activity (or mass fraction) for any given compound is found from eqn (10) by simple application of the chain rule:
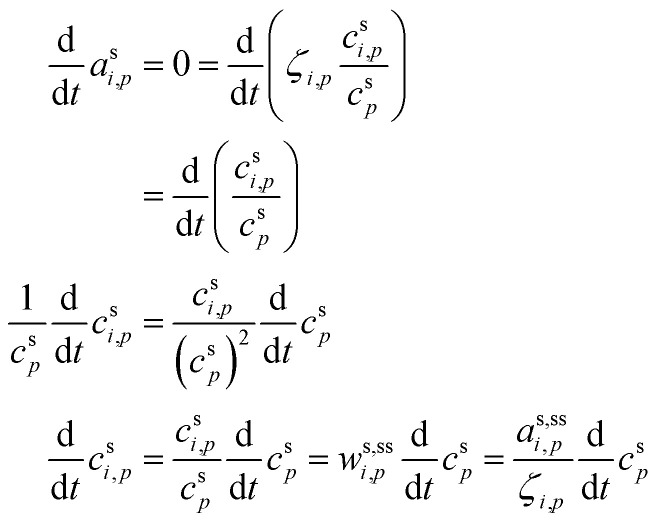


The suspended concentration is the total quantity in suspension per volume of air, so it is affected by the condensation flux in that volume, but also any condensed (suspended) phase chemical production or loss from eqn (27):35
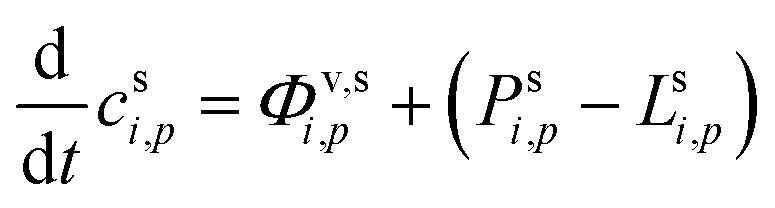


Changes to the total suspended (mass) concentration are only from net condensation, because chemical changes within the condensed phase and thus to the condensed-phase composition do not (immediately and directly) affect the condensed-phase mass:36
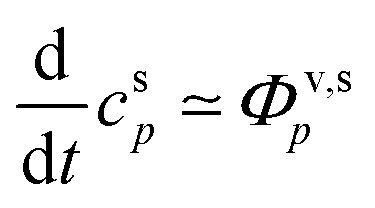


This is only strictly true when using mass concentration units, as association reactions will change the total number of molecules in a particle and thus the mole fractions; this is yet another reason to favor mass concentration.

In some cases it is also useful to split net condensation into a condensation (forward) and evaporation (reverse) term.37
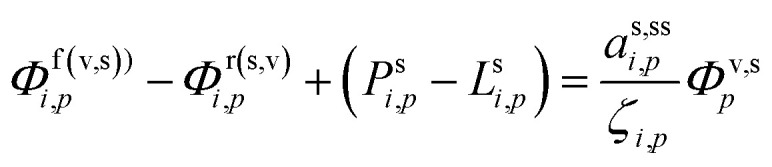


We can solve for the steady-state activity.
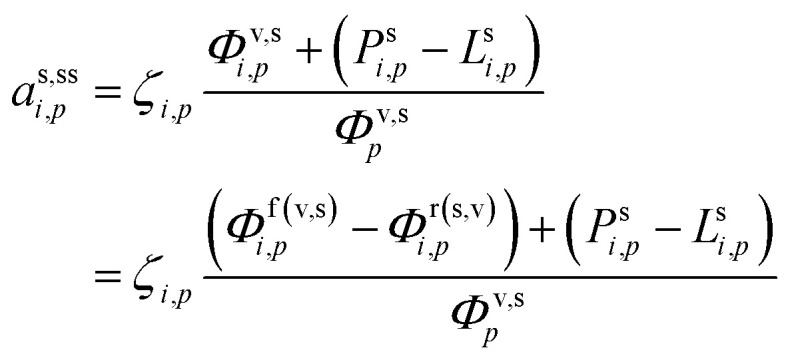


We can now progressively move from the total flux per unit volume of air, *Φ*, to the rates per unit particle surface area, *ϕ*, and also from the total chemical rate, *P* − *L*, to the effective chemical rate per unit surface area, *p* − *l*, by applying the volume to surface area ratio, *d*_*p*_/6:38
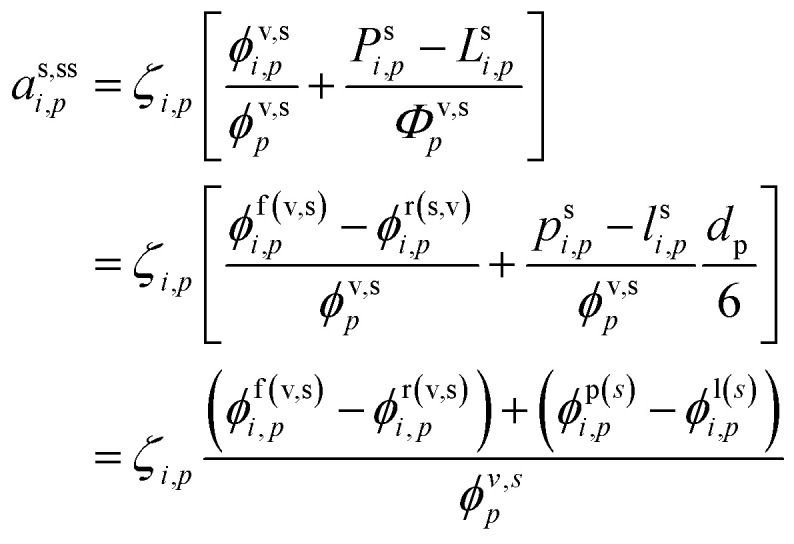
In the last step we express the steady-state activity in terms of both net condensation and effective surface fluxes for production and loss (*Φ*^p(s)^_*i*,*p*_ = *p*^s^_*i*,*p*_*d*_*p*_/6). A flux of external constituents into particles (causing them to grow) works to dilute existing internal constituents – thus decreasing their activity, but the factor of *d*_*p*_ reflects the progressively diminishing influence of surface processes (including fluxes) in larger particles. However, it is always possible to solve for steady-state (constant) activity that will occur when all overall fluxes (and rates) balance.

If the particle composition remains constant (the activities of all species stay the same) while particles grow, then there must be a corresponding net flux of each species to the suspended particle phase, including net condensation and net chemical production, *ϕ*^s^_*i*,*p*_. This is the material flux that drives growth, and it is shown by splitting eqn (38) into a pair of equations.39
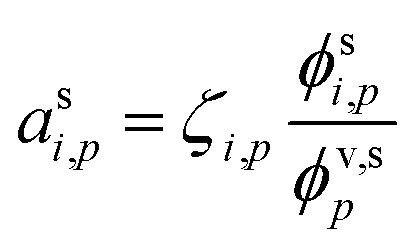

40 *ϕ*^s^_*i*,*p*_ = (*ϕ*^f(v,s)^_*i*,*p*_ − *ϕ*^r(s,v)^_*i*,*p*_) + (*ϕ*^p(s)^_*i*,*p*_ − *ϕ*^l(s)^_*i*,*p*_)

It is important to note that the net fluxes can be either positive or negative, with positive always being the flux to the particle, while the unidirectional fluxes are positive, with the sign explicit in the formulas.

We can now derive expressions for limiting cases isolating various key processes and also explore the features of simultaneous observations of the gas and condensed-phase concentrations for a known steady state growth rate, using the equilibrium dVBS space shown in Fig. 1 but modified to reflect that growth.

### 6.1 Non-reactive condensation

An important case of steady-state growth is non-reactive condensation, with *ϕ*^p(s)^_*i*,*p*_ = *ϕ*^l(s)^_*i*,*p*_ = 0. Further, we shall consider an ideal solution, with *ζ*_*i*,*p*_ = 1 and *s*^⊥^_*i*,*p*_ = const. The particle activity is now the mass fraction, *w*_*i*,*p*_. If the particle composition is constant, then the amount of each species in the particle phase is proportional to its flux. Re-evaporation of volatile species may reduce their fraction in the particles, appearing as a reduced uptake coefficient (*γ*_*i*,*p*_ < 1). Thus, from eqn (39):41
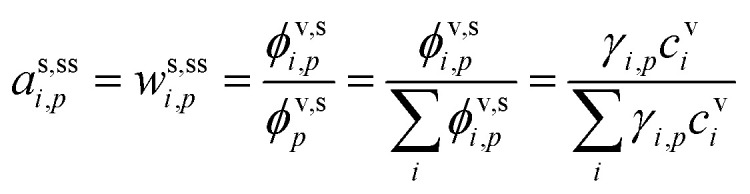
42
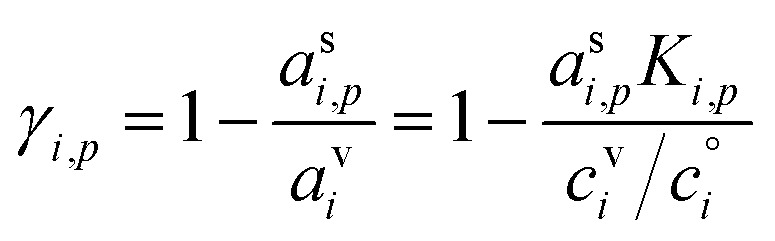


Given two species, the ratio of their condensed (suspended) phase activities is43
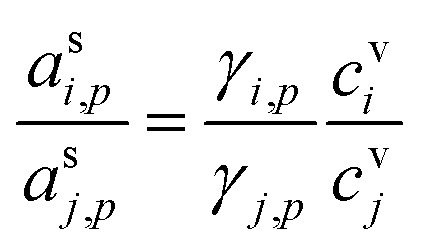


If the excess vapor concentration, *c*^xs^_*p*_, and thus the growth rate, *R*^gr^_*p*_, is known, then we can relate the condensed-phase activity of the species and the vapor concentration using eqn (23):44
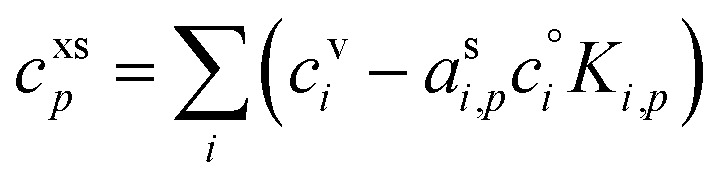
45
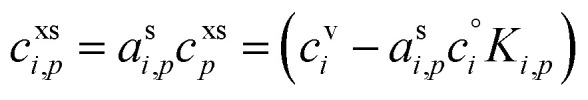
46
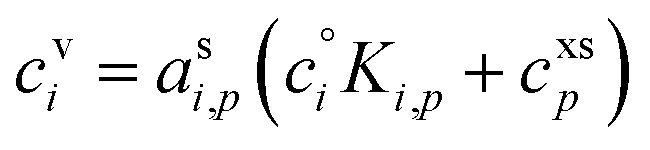
47
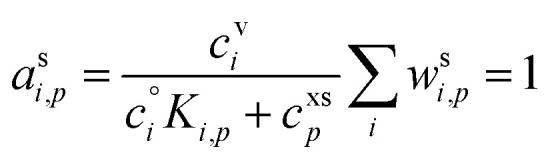
48
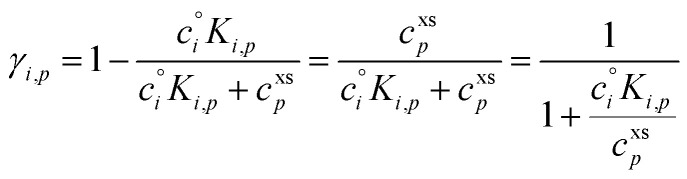


This establishes a minimum vapor concentration for any given observed condensed phase activity, assuming non-reactive condensation is the only significant process.

49 *c*^v^_*i*,min_ = *a*^s^_*i*_*c*^xs^_*p*_

Eqn (49) defines the limiting diagonal of the dVBS. It states, for a given observed growth rate and thus a given effective condensible vapor concentration, there is an excluded region of the activity phase space. Assuming there are no reactions in the particles, the growth is (and was always) at steady state, and a species has a high mass fraction in those particles, then that species must have a correspondingly high concentration in the gas phase. Conversely, appearance in this infeasible region with high mass fraction and low vapor concentration would thus indicate significant reactions and production in the particles. The region is “infeasible” because it cannot be reached *via* non-reactive condensation alone if the system has been at steady state since inception.

#### 6.1.1 dVBS graph

**Fig. 2:**
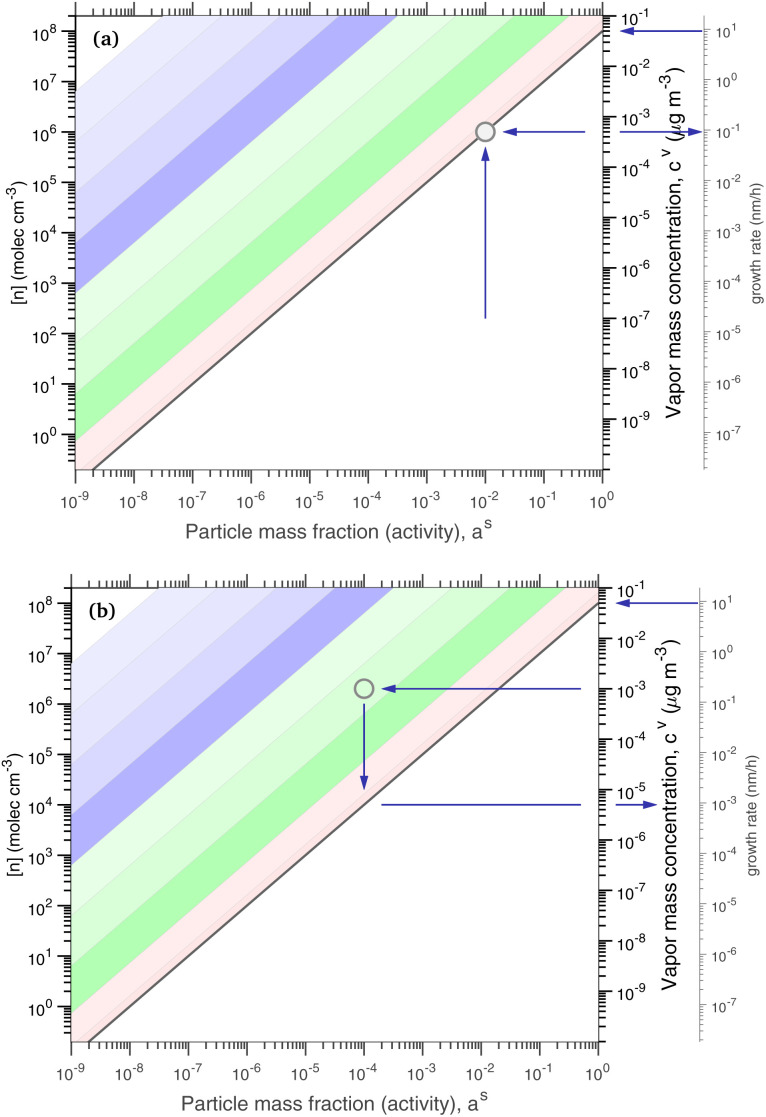
A 10 nm h^−1^ diagonal volatility basis set (dVBS) for saturated and unsaturated vapors. (a) A quasi non-volatile ELVOC condensing almost irreversibly. (b) A semi-volatile SVOC condensing reversibly to near equilibrium. These are steady-state phase diagrams of vapor mass concentration (*c*^v^_*i*_) *vs.* condensed (suspended) phase mass fraction (activity, *a*^s^_*i*_) for organic particle growth with *c*^xs^ = 0.05 μg m^−3^ driving (*d*_*p*_ = 10 nm) growth rates near 10 nm h^−1^, as indicated with the tertiary *y*-axis and an arrow pointing from the growth rate. Growth is driven from the gas phase, so for a given growth rate a given vapor concentration (and volatility), it will sustain a given steady-state mass fraction (or a given mass fraction will require a given vapor concentration). In panel (a), the (gray) ELVOC with saturation concentration 
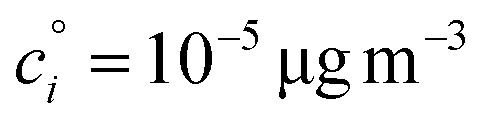
 and particle mass fraction *a*^s^_*i*_ = 0.01 (identical to Fig. 1) is displaced as shown by the vertical arrow from an equilibrium vapor mass concentration *c*^v^_*i*_ = 10^−7^ μg m^−3^ to a sustained vapor mass concentration *c*^xs^_*i*_ ≃ *c*^v^_*i*_ = 5 × 10^−4^ μg m^−3^ (shown as a gray circle), contributing 0.1 nm h^−1^ to the growth rate (indicated with paired horizontal arrows extending from the *c*^v^ axis). In panel (b), the (green) SVOC with saturation concentration 
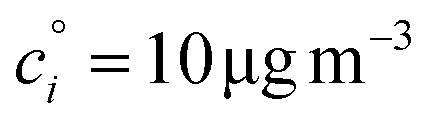
 and condensed phase mass fraction *a*^s^_*i*_ = 10^−4^ is imperceptibly displaced from an equilibrium vapor mass concentration *c*^v^_*i*_ = 10^−4^ μg m^−3^ because the excess vapor mass concentration *c*^xs^_*i*_ = 5 × 10^−6^ μg m^−3^ is a small fraction of the equilibrium value. Although the vapor concentration is similar to the ELVOC, the excess concentration is two orders of magnitude lower and it contributes just 10^−3^ nm h^−1^ to the growth rate.

The constraints as well as key diagnostic features of this steady growth with non-reactive condensation are shown in Fig. 2. Here we consider a typical (relatively fast) growth rate of 10 nm h^−1^.^9,10,41^ First, the growth rate establishes the total excess concentration, as shown in eqn (8) and (25). We add a third *y*-axis with growth rate to the diagnostic plot, with *c*^v^ = *c*^xs^ for *a*^s^ = 1. This growth rate (or the associated excess vapor concentration) establishes the name for the dVBS, so this is a 10 nm per h dVBS, and the condensation limiting (black) diagonal line is given by eqn (49). This is shown in both panels with the upper horizontal blue arrow connecting the growth rate axis to the vapor mass concentration axis.

Like the equilibrium case in Fig. 1, the diagonal dVBS bands (*i* → *b*) are now defined for each volatility 
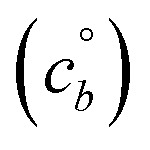
 by eqn (47), with the delimiting lines for 
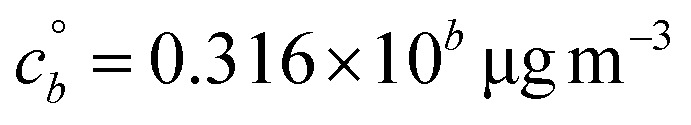
; *b* = {−12…7} (*i.e.* centered on 10^*b*^) and the standard color scheme. Growth has an imperceptible effect on higher volatility bands 
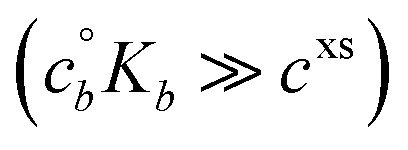
, but all the lower volatility bands 
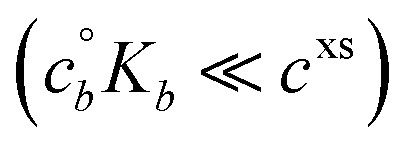
 collapse onto the condensation line defined by eqn (49). Other than a narrow range 
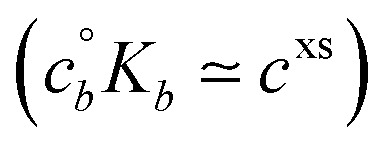
, non-reactive species are almost entirely either quasi-irreversible, in which case they appear along the limiting line, or they are quasi-equilibrated, in which case they appear in the “proper color band” of the equilibrium VBS shown in Fig. 1 (in reality the vapor concentration is slightly higher than the equilibrium value to sustain the necessary excess, but for relatively volatile species this is almost imperceptible).

Fig. 2a shows the same example ELVOC species from Fig. 1 for a case with *c*^xs^ = 5 × 10^−2^ μg m^−3^ driving growth at *R*^gr^_10_ ≃ 10 nm h^−1^. The gray symbol color indicates ELVOC volatility. The ELVOC has the same particle mass fraction as in Fig. 1 (*a*^s^_*i*,*p*_ = 0.01); however, that now means this ELVOC is driving 1% of the growth, or 0.1 nm h^−1^, and thus requires a much higher vapor concentration. This is shown with horizontal blue arrows extending to the symbol and growth rate from the right-hand *y*-axis. The vapor concentration compared to equilibrium is enhanced by a factor of 5000, shown with the vertical blue arrow extending from the equilibrium location to the symbol. Because 
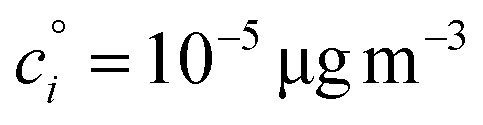
 ≪ *c*^xs^ = 5 × 10^−2^ μg m^−3^, the ELVOC lies on the quasi-irreversible limit line for non-reactive condensation.

Fig. 2b shows reversible semi-volatile condensation for an SVOC with 
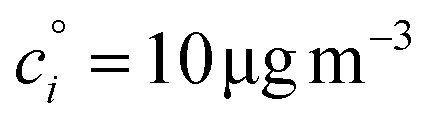
 and *c*^v^_*i*_ = 10^−3^ μg m^−3^ that is barely perturbed from its equilibrium, for the same overall growth as Fig. 2a. The green symbol color indicates SVOC volatility. In this case, 
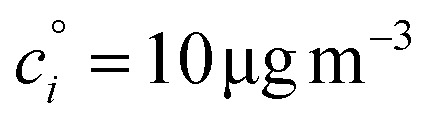
 ≫ *c*^xs^ = 5 × 10^−2^ μg m^−3^, so the SVOC lies near the equilibrium location with a slight excess sustaining its contribution to growth. The vapor concentration locates the SVOC in the *y*-coordinate, with the *x*-coordinate, the mass fraction, *w*_*i*,*p*_, given *via*eqn (46); this is shown in the figure with a left-facing arrow ending at the symbol. The uptake coefficient rom eqn (48) is *γ* = 5 × 10^−3^. The excess concentration is a small fraction of the vapor concentration; this is given by eqn (45). The excess concentration is found visually by extending a vertical (vertical arrow) to the condensation line, and then projecting back to the *y*-axis as shown, giving *c*^xs^_*i*_ = 5 × 10^−6^ μg m^−3^. This partial growth of 10^−3^ nm h^−1^ is roughly 10^−4^ of the total growth and so the SVOC has a mass fraction *w*_*i*_ = *a*^s^_*i*_ = 10^−4^.

**Fig. 3:**
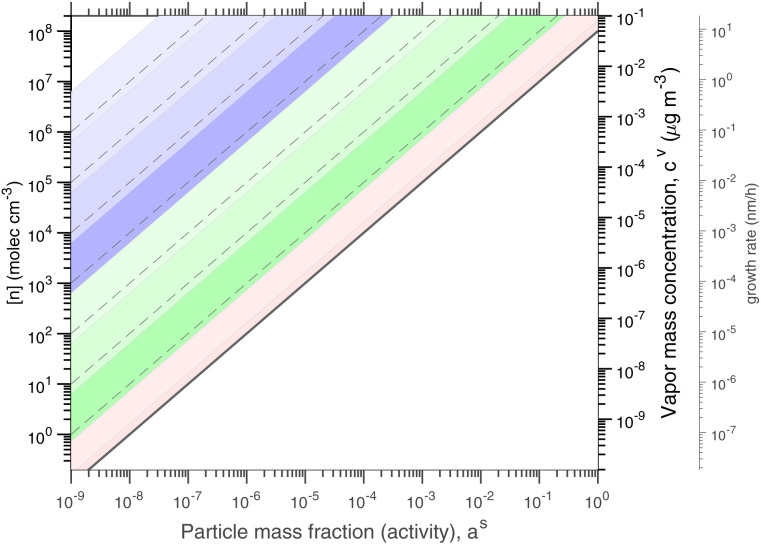
A 10 nm h^−1^ dVBS with uptake coefficient lines. Species with volatility 
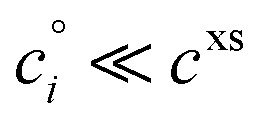
 condense quasi-irreversibly along the solid *γ*_*i*,*p*_ = 1 condensation line defined by the growth rate, whereas species with 
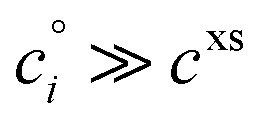
 remain in quasi-equilibrium. The dashed diagonal lines parallel to the condensation line indicate condensation uptake coefficients (*γ*_*i*,*p*_ = 0.1, 0.01, …) for these still volatile species. The quasi non-volatile portions of the dVBS space collapse onto the *γ* = 1 line, leaving a white infeasible region where a pure condensation steady state cannot exist – a species cannot appear *via* condensation in a rapidly growing particle at a high mass fraction without a sufficiently high vapor concentration to sustain it.

The general phase space for this 10 nm per h dVBS is illustrated in Fig. 3, with uptake coefficients in the semi-volatile (relatively unperturbed) region (
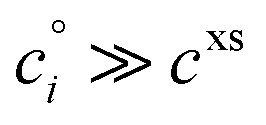
, neglecting *K*) shown as dashed lines parallel to the *γ* = 1 limiting line in the log–log space for each decade (*γ* = 0.1, 0.01…). All the quasi non-volatile VBS bins (
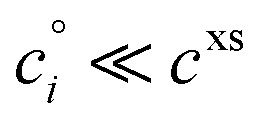
) collapse onto the diagonal *γ* = 1 limiting line (the “condensation limit”), leaving an infeasible region for purely condensation driven particle growth and composition. All the colors for volatilities lower than the salmon colored LVOC appear on the figure, but within the narrow range of the diagonal, black condensation limit line. This white infeasible region in the lower right Fig. 3 is a key diagnostic feature – species appearing in that region, with high condensed-phase fractions but low gas-phase concentrations cannot have arisen in the particles due to condensation, and so must be formed *via* chemistry within the particles.

#### 6.1.2 Constraints on composition

If the condensed-phase activities (mass fractions) are known for a given excess concentration (growth rate), the steady-state non-reactive uptake can be solved for the gas-phase (vapor) concentrations that must be driving that growth. However, the constraint on the total condensed-phase mass fraction adding to unity can also be applied to determine the growth rate and condensed phase activities for a given set of known (constant) gas phase concentrations.
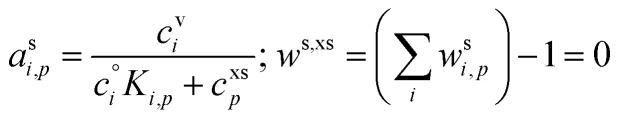
Specifically, the total excess mass fraction can be treated as a function of *c*^xs^_*p*_, followed by a straightforward root finding to determine the proper *c*^xs^_*p*_ (analogous to finding the equilibrium mass in the classical VBS^16^).

#### 6.1.3 Defining characteristics

The defining features of (constant) non-reactive uptake for the two subtypes are as follows:

(1) Quasi irreversible (non-volatile) species fall on the limiting line defined by the growth rate, with relative particle mass fraction proportional to relative gas-phase concentration.

(2) Quasi equilibrium species fall in the equilibrium phase space, with slightly lower mass fractions than expected at equilibrium to sustain the excess vapor activity for growth.

Under most circumstances the growth is governed by the quasi irreversible fraction of the condensing species, with the quasi equilibrated species simply serving as a multiplier for the growth. A partial growth rate will be determined by the total concentration of quasi non-volatile vapors, and then the overall activity of the semi-volatiles will define a multiplier for this rate. This applies to all condensing species, including water, so for example the water activity in the growing particles will be sustained at the relative humidity, and if this causes the water volume fraction to be, *e.g.* 0.2, then the growth will be 20% faster than for dry conditions. This can also be constrained by hygroscopic growth factor measurements. The extra water would also influence condensation by increasing the true particle surface area, meaning the actual composition (not that observed after drying samples, for example) should be used for these diagnostics. The specific issue of water is also a complication if samples are dried during measurement.

### 6.2 Irreversible reactive uptake

For reactive uptake with a steady-state solution, the condensing species (monomer, *m*) must react to form something (here we call this a dimer, *d*). For the purposes of illustration we will assume that the product is otherwise absent from the system and so is unique. The total uptake coefficient must be larger than the non-reactive condensation uptake coefficient if uptake is irreversible, so *γ*_*m*,*p*_ > *γ*^nr^_*m*,*p*_. The consequences of reactive uptake will depend on whether the product is more or less volatile than the condensing species. Though the nomenclature “dimer” implies that the product is less volatile than a (potentially quite volatile) monomer, the reverse case can be true as well.

The steady-state flux balance (eqn (38)) is still driven by the (observed and constrained) monomer vapor concentration, but includes loss of the monomer in the particles, corresponding production of the dimer, growth from the dimer, but also at least some evaporation of the dimer and the associated concentration balance of the dimer vapor. This last balance unavoidably involves the bulk aerosol loading (the condensation sink) as well as whatever bulk loss process exist for the dimer in either phase (in an experiment this will typically be wall and ventilation loss). Rather than specifying a condensed phase rate coefficient, we specify a reactive uptake coefficient, *γ*^rx^_*m*,*p*_. Some of the monomer will remain in the particles as well, giving an overall uptake coefficient, *γ*_*m*,*p*_ = *γ*^rx^_*m*,*p*_ + *γ*^m^_*m*,*p*_.

The flux expressions for the monomer include no chemical production, with the net flux of monomers to the suspended particles that remain in the particles as monomers, *ϕ*^s^_*m*,*p*_, given by eqn (40).50
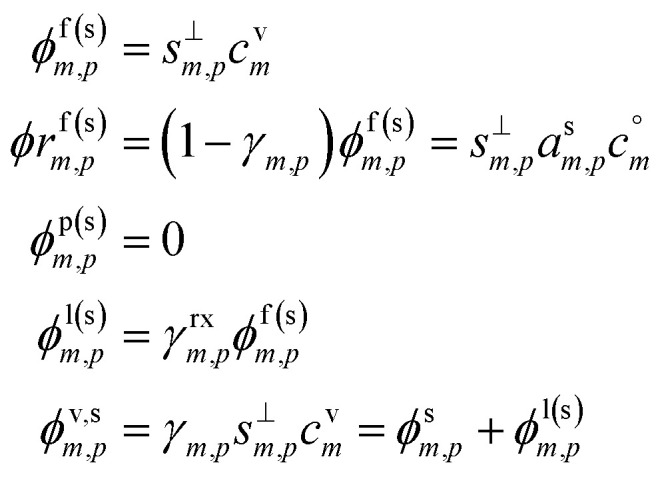
From eqn (50) we see that the net flux of monomers to the particles is split between the flux that remains in monomer form and the flux that is lost (to dimers). The corresponding flux expressions for the dimer include a net (evaporation) flux that must be balanced by all losses of vapors, which we represent as wall loss, *k*^w^_*d*_. We can thus find the net flux of products that remain in the particle, *ϕ*^s^_*d*,*p*_.51
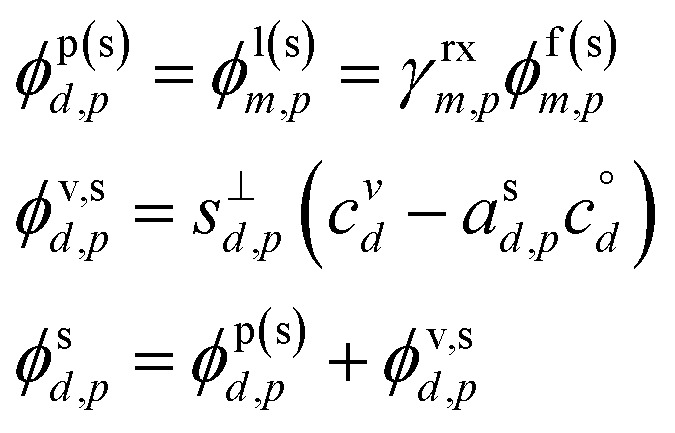
52
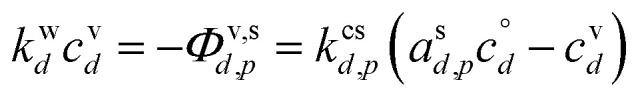


To achieve a flux balance with greater net uptake than the non-reactive case, the evaporation (reverse) flux of the monomer must be reduced as a fraction of the forward flux from (1 − *γ*^nr^_*m*,*p*_) to (1 − *γ*_*m*,*p*_). This is a direct consequence of the (monomer) activity in the particle (on the surface), and so the activity will be reduced relative to the non-reactive steady-state value.53
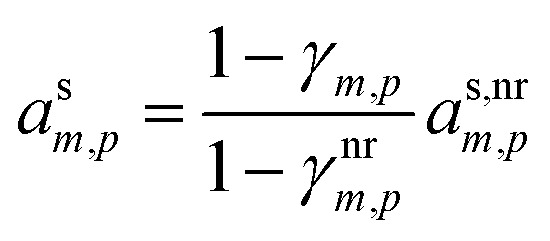


To be sustained, this will still lead to some condensational growth from the monomer directly, given by the net monomer flux, *ϕ*^s^_*m*,*p*_, because the monomer activity in the growing particle will remain at this non-zero steady state.

54 *ϕ*^s^_*m*,*p*_ = *s*^⊥^_*m*,*p*_*a*^s^_*m*,*p*_*c*^xs^_*p*_

55 *γ*^m^_*m*,*p*_ = (1 − *γ*_*m*,*p*_)*γ*^nr^_*m*,*p*_

The rest of the growth will be driven by the reactive uptake.

56 *γ*^rx^_*m*,*p*_ = *γ*_*m*,*p*_ − *γ*^m^_*m*,*p*_

This reactive flux will be balanced by formation of the product (dimer), which will in turn lead to some product evaporation and thus a non-zero product vapor concentration; if the product is volatile, “dimer” may be exchanged for “desorber”. From eqn (51) and (40), with a common collision speed (*s*^⊥^_*m*,*p*_ = *s*^⊥^_*d*,*p*_ = *s*^⊥^_*e*,*p*_):
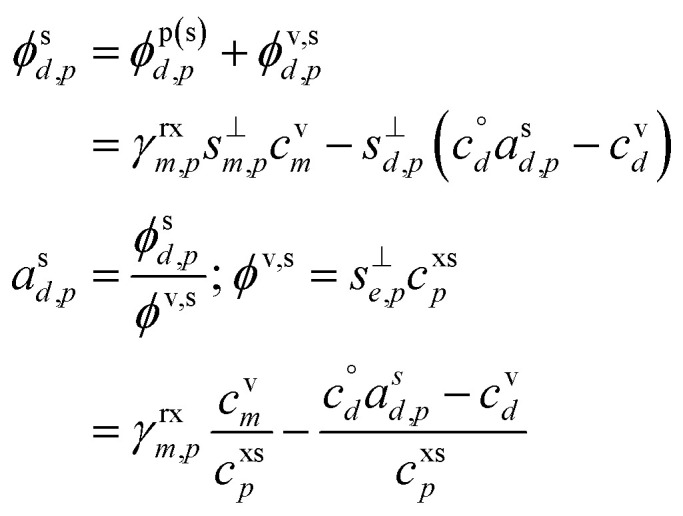


Rearranging terms we find a useful expression for the activity of the product.57
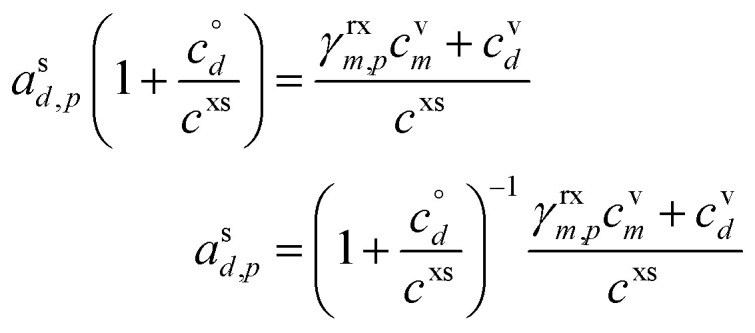


From eqn (52) we can find the vapor concentration of the product.58
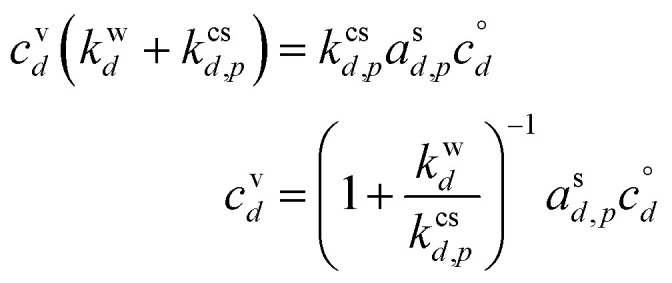
Whereas for non-reactive uptake and a given particle phase activity the vapor concentration is always higher than at equilibrium, the reverse is true here. Because growth is driven by excess vapor, when a product is formed in the condensed phase the resulting product vapor concentration is always lower than at equilibrium. Depending on the relative magnitude of the vapor sinks and the condensation sink, shown in eqn (58), the product vapor concentration may be far below equilibrium. It is unlikely that this product would be observed at high concentration in the vapor phase (even if it were quite volatile) unless the total condensation sink of the suspended particles was quite high compared to other losses such as wall losses.^42^

#### 6.2.1 Limiting cases

There are two different pairs of limiting cases for reactive uptake. The first is characterized by the relative volatility of the product. The second is characterized by the relative magnitude of the condensation sink compared to the other vapor losses.

A low volatility (dimer) product has 
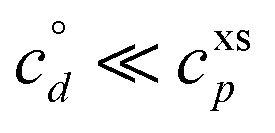
. In this limit there is negligible evaporation of the product, which will have a very small vapor concentration. We also assume that the product evaporation has a negligible effect on the product activity.59
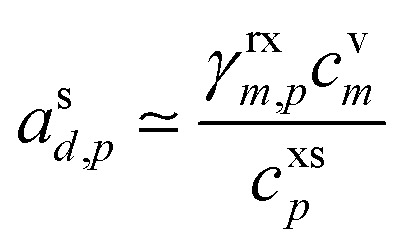


A volatile (desorbing) product on the other hand has 
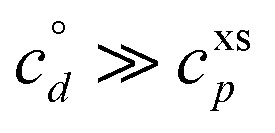
.60
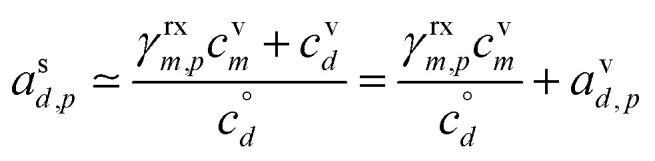


For a weak condensation sink, *k*^cs^_*d*,*p*_ ≪ *k*^w^_*d*_, and the presence of particles will have a negligible effect on the vapor concentrations, with the sole exception of the product.61
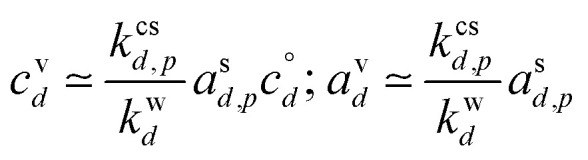


Coupled with a volatile product, this also gives62
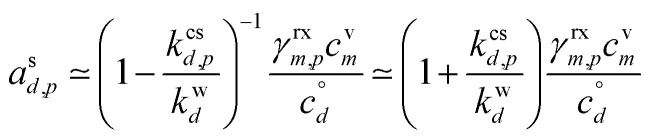


However, for a strong condensation sink, *k*^cs^_*d*,*p*_ ≫ *k*^I^_*d*,*p*_, and particle-vapor equilibrium will prevail.
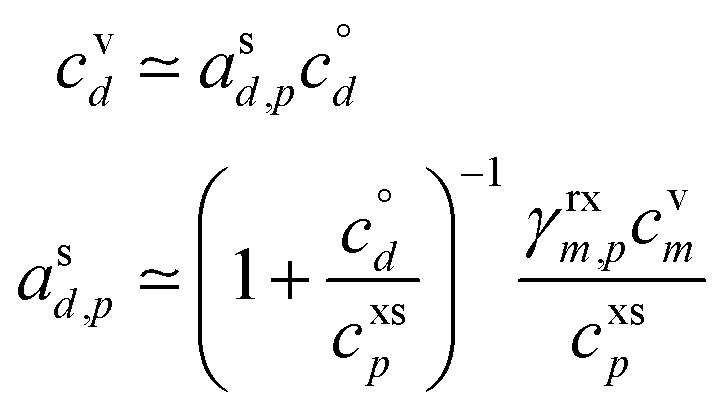


The product volatility determines what fraction remains in the particles and what fraction evaporates to be lost to the (comparatively slow) vapor loss processes.

#### 6.2.2 dVBS graph for a low volatility product

When a low volatility product (dimer) is formed (and observed), the effect is dramatic. The products appear in the otherwise infeasible region, and they can appear anywhere within that region, limited only by the relative abundance of the monomer. The diagnostic is highly effective because the more important this reactive uptake is to overall growth, the farther into the infeasible region the product will appear, with high particle mass fraction and low vapor concentration. To be noteworthy, the product particle activity should be large (thus representing a large fraction of the overall growth). Simply put, if reactive uptake drives a lot of growth, then there will be a lot of the product in the particles and virtually none in the gas phase.

**Fig. 4:**
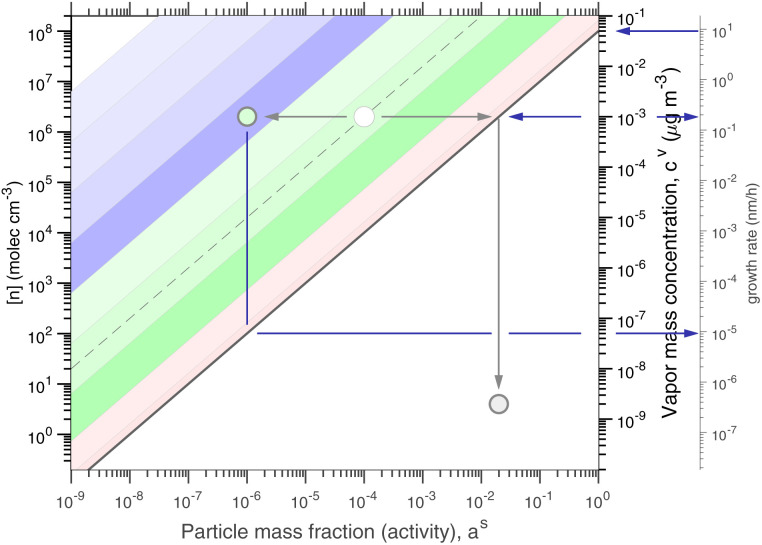
A 10 nm h^−1^ dVBS showing reactive uptake. Condensation of an SVOC with saturation concentration *c*° = 10 μg m^−3^ and vapor concentration *c*^v^ = 10^−3^ μg m^−3^, which would normally appear with a mass fraction *a*^s^_*i*_ = 10^−4^, indicated with a white (vacant) circle. This would correspond to *γ*_*i*_ = 5 × 10^−3^, which is indicated with a dashed diagonal line. Reactive uptake with *γ*_*i*_ ≃ 0.99 drives the SVOC activity down to 10^−6^, plotted with a green circle indicating the SVOC saturation concentration. This green SVOC is now out of color order, appearing in the blue IVOC dVBS band. The reactive uptake forms an ELVOC species with *c*° = 10^−6^ μg m^−3^, and the ELVOC appears with *a*^s^_*i*_ = 0.02, which is the maximum possible sustained by condensation at *c*^v^ = 10^−3^ μg m^−3^. Because the ELVOC is formed in the condensed phase, its subsequent vapor concentration is at most the equilibrium value of only *c*^v^ = 2 × 10^−8^ μg m^−3^. However, in many cases the aerosol condensation sink is lower than other vapor losses (*e.g.* wall losses). If the condensation sink is 10% of the other vapor losses, this ELVOC reaches a steady state of *c*^v^ = 2 × 10^−9^ μg m^−3^, plotted in ULVOC gray well within the infeasible region. This region is infeasible for non-reactive condensation, but populated by reactive uptake.

Fig. 4 shows an example for condensation of an SVOC at *c*^v^_*i*_ = 10^−3^ μg m^−3^ with *γ*_*i*_ = 0.99 creating a ULVOC. The SVOC would normally be found in the green diagonal, shown here with an empty symbol, with modest uptake (*γ*^nr^ = 5 × 10^−3^) indicated with the dashed diagonal line. The very fast reaction produces a ULVOC dimer and depletes the monomer activity by roughly a factor of 100, shifting it to the left in the *x* direction towards lower particle mass fraction. The maximum mass fraction of the ULVOC dimer is given by the horizontal value of the condensation diagonal at the SVOC vapor concentration, as shown with the arrow extending from the open symbol to the condensation limit diagonal. Overall, the uptake coefficient of the SVOC increases by a factor of 200. The total growth rate driven by the SVOC uptake is 0.2 nm h^−1^, with a modest 10^−5^ nm h^−1^ ultimately driven by the SVOC and the rest by the ULVOC. Both these contributions to growth are indicated with arrows extending to the growth rate *y*-axis to the far right. In this example, as the SVOC monomer is substantially depleted; it is shifted to a much lower particle activity and so appears “out of color order” as a green symbol in the blue IVOC region. However, it is the dimer product ULVOC that jumps out. It appears near the equilibrium location for a ULVOC in the particles at high activity but lower by a factor of 10 in this example because the condensation sink is assumed to be ten times lower than the other vapor sinks. It is thus well and truly within the infeasible region for non-reactive condensation. Compounds in this region, with high particle phase activity yet very low corresponding vapor concentration, are unequivocal evidence for particle-phase formation chemistry during steady-state growth experiments.

The sense of this interaction is shown with arrows in Fig. 4. The monomer vapor at a fairly high concentration (*y* = 10^−3^ μg m^−3^) would appear with a modest mass fraction of 10^−4^ were it not reactive, shown with the open circle. However, its collisions with the particles could drive growth of 0.2 nm h^−1^ with rapid uptake (shown with the right-facing horizontal arrow, or 2% of the overall 10 nm h^−1^ growth. This would require (and result in) a product (dimer) comprising 2% of the particle mass, which would appear as the gray circle deep in the infeasible region, given a low volatility. Because this uptake would also deplete the monomer in the particles, the monomer mass fraction would drop, in this case to 10^−6^ as shown with the filled green circle. For this example we assume that condensation is a minor sink for the vapor (*e.g.* when wall loss dominates during an experiment) and so the shift from the non-reactive (open circle) to the reactive (green circle) is horizontal. While the “out of color order” shift of the vapor might be difficult to observe amid a sea of vapors, the product (if observable) would stand out easily in the infeasible region.

#### 6.2.3 dVBS graph for a volatile product

**Fig. 5:**
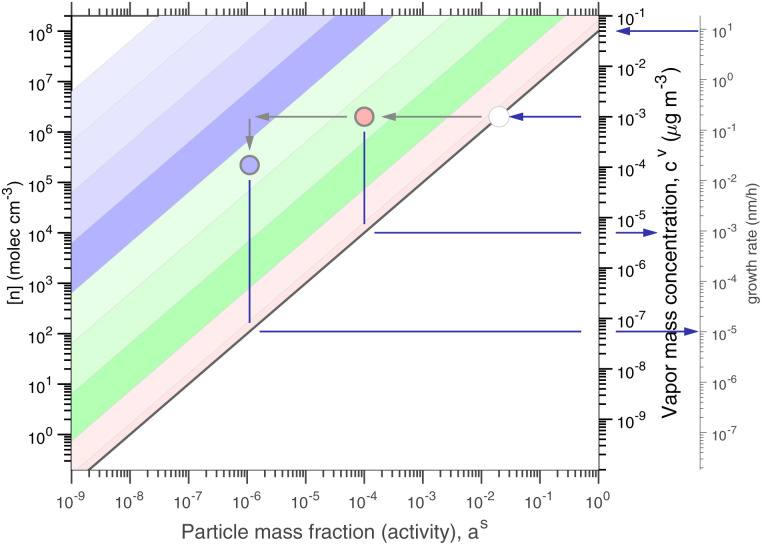
A 10 nm h^−1^ dVBS showing “rejected” reactive uptake of a low volatility species. Reactive uptake of an LVOC (salmon) species yields a more volatile IVOC (blue) product. This slows the growth rate compared to the expected non-reactive uptake. Instead of appearing on the limiting line, the LVOC has a substantially lower particle phase activity because of its reactive loss, appearing as the salmon circle at low particle mass fraction instead of the empty circle at higher particle mass fraction. The volatile (blue IVOC) product appears in the gas phase but with a lower-than-equilibrium gas-phase concentration determined by the ratio of the condensation sink to other vapor losses. Thus both parent and child species appear “out of color order”.

If instead a low-volatility monomer species reacts heterogeneously to produce a more volatile product, something of the reverse effect occurs; this is shown in Fig. 5. Here an LVOC monomer, again at *c*^v^_*i*_ = 10^−3^ μg m^−3^, condenses, but instead of appearing at the condensation limit line, more than 99% reacts to yield an IVOC product and so the contribution of that LVOC to growth is greatly reduced. The overall growth is reduced by about 2%, which this LVOC condensation would otherwise have provided. Both the LVOC monomer and the IVOC product appear with some activity in the particles, but both are “out of color order” in their location. The LVOC is out of color order because it is depleted from the particle and thus displaced leftward in *x* as shown by the salmon colored circle in the green diagonal; the IVOC is out of color order because the condensation sink is too low to sustain an equilibrium vapor concentration and so it is displaced downward in *y* as shown by the blue circle in the light green diagonal. These mis-placed species are again characteristic of this heterogeneous loss, but only the misplaced LVOC is likely to be evident in data as a species that “should” have a high particle mass fraction yet is actually less abundant.

The case of a comparatively high condensation sink is not consistent with the initial assumption of constant (measured) vapor concentrations and constant, steady-state particle activities. The high condensation sink case is more consistent with a chamber mass balance experiment focused on Secondary Organic Aerosol (SOA) mass yields, where it is desirable to have a high condensation sink and thus render vapor-wall (or ventilation) terms secondary.^43,44^ On the other hand, the low condensation sink case is preferable for nucleation and growth experiments, provided that the vapors can indeed be measured. For the high condensation sink case, it is more likely that the vapor production terms, *P*^v^_*i*_, will remain constant, and the actual vapor concentrations will evolve as the condensation sink (and overall aerosol mass) grows. This will affect different species differently. Relatively volatile species will establish a gas–particle equilibrium and so the overall flux balance for vapors will still be *P*^v^_*i*_ = *L*^v^_*i*_, thus sustaining a constant vapor concentration. However, low volatility species will be almost irreversibly lost to the growing particles, and so their vapor concentration will steadily drop as the condensation sink rises. Because of this, the particle composition will evolve as the particles grow, with more volatile species being progressively more abundant in the particles, consistent with the well-established VBS analysis of partitioning in chamber experiments.^16,45^

#### 6.2.4 Overall effect on growth

We are interested in the extent to which reactive uptake enhances (or retards) growth, which depends on the degree to which condensation of the reacting species would drive growth in the first place. If reactive uptake enhances growth, it will do so by increasing the overall *γ*_*i*_ and forming a less volatile product where uptake would otherwise have been modest. The monomer itself will be depleted in the particles, but this will only be substantial if conversion to the product (dimer) is nearly complete. If, on the other hand, reactive uptake retards growth, this requires that an otherwise low volatility (monomer) species react to form a much more volatile product (“desorber”); here the reaction must deplete the monomer in the particles for the reaction to cause significant retardation.

The overall effect on growth is given by the ratio of the total activity (mass fraction) of the monomer and dimer (or desorber) to the non-reactive monomer activity, for a gain of63
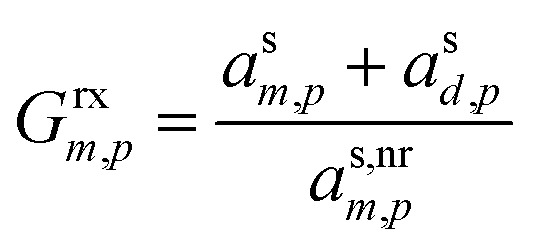


Giving a growth enhancement of64
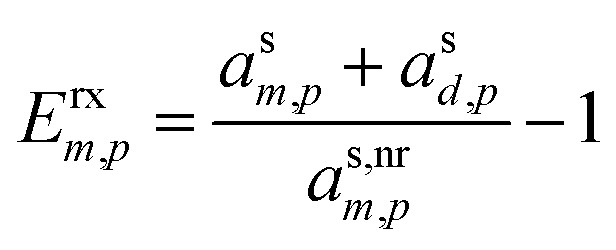


This ranges from very large (a substantial enhancement) to −1 (100% retardation). For the examples in Fig. 4 and 5, the gain factors are 20 and 0.01 and the enhancements are 19 and −0.99, respectively.

#### 6.2.5 Defining characteristics

Irreversible reactive uptake leaves two distinctive signatures on the combined gas and particle phase data, depending on whether the product is effectively non volatile or relatively volatile:

• For non-volatile products, the product appears in the infeasible region of the gas and particle composition phase space, with the reactive condensing vapor depleted from the particles and thus shifted from its expected location towards lower activity and thus higher equilibrium volatility color.

• For volatile products, both the product and the condensing vapor are shifted towards lower particle activity and again are out of color order.

• The contribution to growth of the condensed-phase reaction depends on the sense of this “disorder” from the condensing vapor:

– If the condensing vapor has relatively high volatility and the product has low volatility and appears in the infeasible region, then the reaction accelerates growth.

– If the condensing vapor has relatively low volatility and should appear on the condensation limit line, and the product is low volatility and in the infeasible region, then the reaction has little or no effect on growth.

– If the condensing vapor has relatively low volatility and should appear on the condensation limit line, and the product is volatile, then the reaction retards growth.

### 6.3 Thermally reversible reactive uptake

The diagnostics presented thus far are compelling, but a contingency is always “if measured”. Specifically, they require accurate gas-phase measurements of all condensing species, and precise measurements of (relative) particle composition, again of all species in the particles as they exist *in situ*. The diagnostics are biased by compounds that are not measured (*i.e.* those that are refractory or insoluble) or destroyed during sampling or measurement. One example is temperature programmed desorption (TPD), used in the FIGAERO chemical ionization mass spectrometer as discussed below.^46^ Compounds that decompose when heated may confound this analysis, but still leave telltale traces.

It is possible that reactive uptake and the associated condensed-phase chemistry will form products with activation energies for decomposition that are lower than the enthalpy of vaporization, meaning that they will decompose before vaporizing during TPD or other thermal desorption measurements. The method then becomes temperature programmed reaction spectroscopy (TPRS). The lower the volatility, the higher the vaporization enthalpy, and the more likely this becomes. If decomposition re-forms the reactant “monomers”, the signals will appear as those monomers; however, the TPD thermogram may then contain multiple peaks.^47^ The lowest temperature peak should represent any unreacted monomer (unless the product is so loosely bound that it dissociates before even the monomer evaporates), but peaks at higher temperature would reflect decomposition of (possibly multiple) reaction products. The dynamics of this process are identical to the dynamics of irreversible dimer formation, but with measurement *via* these additional monomer signals.

#### 6.3.1 dVBS graph for thermally reversible reactive uptake

**Fig. 6:**
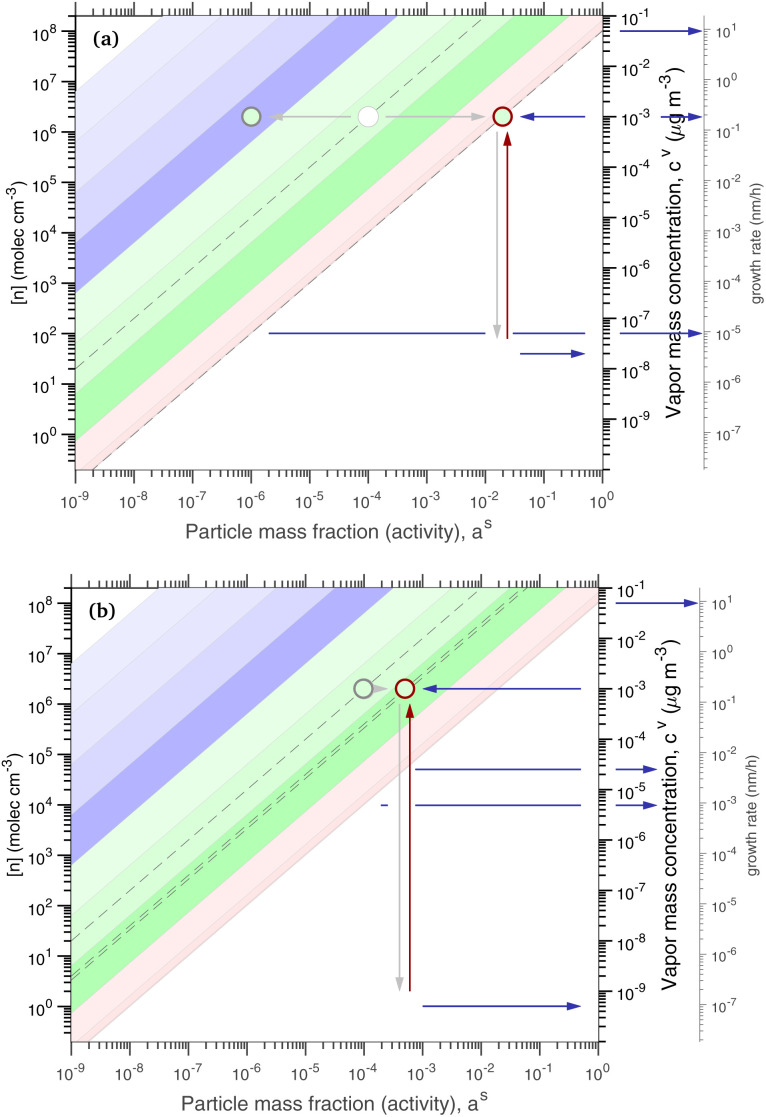
A 10 nm h^−1^ dVBS showing two cases of reversible reactive uptake. Condensation of an SVOC with saturation concentration *c*° = 10 μg m^−3^ and vapor concentration *c*^v^ = 10^−3^ μg m^−3^ would normally appear with a mass fraction *a*^s^_*i*_ = 10^−4^ (and *γ*_*i*_ = 5 × 10^−3^ indicated with a dashed line in the green diagonal band). Instead this results in formation of an ELVOC species, which would normally appear in the infeasible region in the location of the vertical gray arrow. However, thermal dissociation (the vertical red arrow) reforms the SVOC monomer during sampling (*i.e.* heating of particles for vaporization). No ELVOC circle is shown as no signal (of that species) would be observed from the particles. The greater net uptake increases the apparent activity (signal) of the portion of the monomer derived from this reactive uptake and decomposition. This is shown as a red-edged circle to suggest thermal decomposition, with green fill for the SVOC monomer volatility. The portion of the monomer signal associated with unreacted monomer drops if the reaction competes with evaporation. This is shown with a normal (black-edged) green circle. Two examples are shown. (a) Near instantaneous dimerization with *γ*^rx^ = 0.99. (b) Fractional dimerization with a total uptake coefficient of *γ*^tot^ = 0.03 and a reactive uptake coefficient of *γ*^rx^ = 0.025. The particle mass fraction and thus growth is split between components driven by the reactive uptake and the semi-volatile uptake; the growth is shown by the blue arrows pointing at the growth-rate axis. Evaporation of the ELVOC from the particles (without heating) leads to a (very small) ELVOC vapor concentration *via* evaporation, as shown by the horizontal blue arrows extending from where the ELVOC circle would be in the infeasible region, if any signal were observed in the particles.

Fig. 6 shows examples of reversible condensation for an SVOC with 
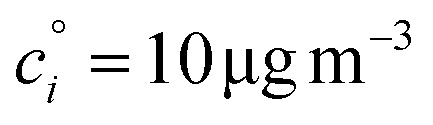
 at a vapor concentration, *c*^v^_*i*_ = 10^−3^ μg m^−3^, creating an ELVOC, for two cases given by two different overall uptake coefficients, *γ* = 1 and *γ* = 0.03. The thermal (decomposition) product is shown as a red-edged circle, and in each case an invisible product is formed (indicated with a vertical gray arrow toward the unseen product in the infeasible region) but then decomposes during measurement (indicated with a reverse red arrow). Some ELVOC would evaporate to the gas phase, here with *y* = 5 × 10^−8^ μg m^−3^; however, this would likely be below the detection limit, and with no measured *x* (particle) value due to the decomposition. Instead, a prominent peak would appear for the SVOC at an unusually high desorption temperature during measurement (low apparent volatility). This is shown with the red-edged green circle at *x* = 0.02 (*i.e.* a high signal).

The rapid uptake case (Fig. 6a) is identical to the irreversible uptake case in Fig. 4, just with a different measured compound (and thus a much higher vapor concentration because the signal is associated with the condensing vapor). In theory, the TPRS thermogram would show two peaks, one (tiny) corresponding to the unreacted monomer and one (large) to the decomposing dimer. The limited uptake case (Fig. 6b) shows the monomer and decomposition symbols closer together, indicating more similar amplitudes (not desorption temperatures) in the TPRS thermogram. This is characteristic – either reversible uptake is nearly complete, and the vapor will appear to have a condensed-phase activity consistent with irreversible uptake on the limiting line (and the monomer peak will vanish almost entirely), or the uptake is partial, in which case the two peaks will both appear in the “semi-volatile” region away from the *γ* = 1 line. In reality, condensing monomers are likely to react with different species, potentially yielding many association products; as these would have a variety of decomposition enthalpies, they would decompose at a range of temperatures, giving TPRS thermograms with multiple peaks. The thermogram might be quite difficult to interpret. In theory, pairs of decomposition peaks might be identified for each invisible association product (an example is oleic acid ozonolysis^47^).

If thermal decomposition were to produce different products than the precursor (monomer), then those products would likely still appear in the infeasible region with high activity in the particles but low vapor signals – unless the product species happened to also be present in the vapor phase due to gas-phase chemistry. Overall, decomposition during measurement can remove signal from the infeasible region that would otherwise characterize particle-phase production, but it would leave a ghost in the form of multiple peaks in a temperature-programmed desorption signal.

It is possible that a condensing monomer could also decompose during thermal desorption, but in this case the original monomer would appear to be depleted in the particles, and the product(s) would be unusually enriched. This is the one case where a false signal would appear in the infeasible region.

## 7 Examples for mixtures

The atmosphere itself, and even most experiments, comprise rich mixtures of organic compounds, and those mixtures are our motivation. The general solution to the steady-state activity will always require iteration, as the activity appears *via* the mass fraction and total mass in a sum in the denominator, and chemical production and loss are also potentially involved. However, several examples are instructive. These include (a) finding the gas concentrations when the particle composition is known and (b) finding the particle composition (and steady-state growth rate) when the vapor concentrations are known. As before, some different processes will influence the overall appearance of the combined particle and vapor phase-space diagrams.

### 7.1 Specified particle composition

For a sequence of illustrations we shall consider a system with 4 major LVOC constituents along with other trace constituents, with a known condensed-phase composition. The LVOC span the range where 
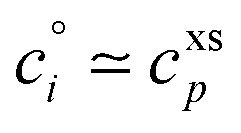
 and so illustrate the major diagnostic features. In this example they have saturation concentrations and activities of
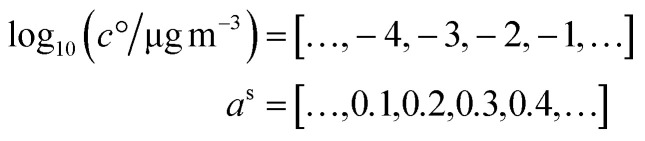


This has features of a typical volatility distribution, with a triangular distribution weighted toward more volatile species in the LVOC range. The sum of the particle activities (mass fractions) is 1.0 as required. Species with lower activity in the particles (*a* < 0.01) span the full range from IVOC through ULVOC; the ULVOC and ELVOC species are minor but quasi irreversible particle constituents, while the SVOC and IVOC species are minor but quasi equilibrated constituents.

#### 7.1.1 Equilibrium mixture

**Fig. 7:**
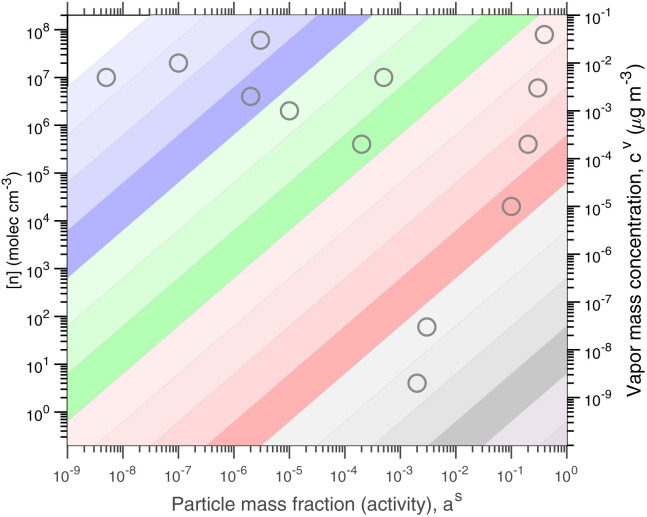
An equilibrium dVBS for a representative volatility distribution, with particle composition dominated by (salmon colored) LVOCs. Vapor activity and condensed-phase (suspended) activity must be equal, resulting in extremely low vapor concentrations of extremely low volatility species (gray shades).

At equilibrium, as discussed above, *a*^v^_*i*_ = *a*^s^_*i*,*p*_ (for all *p*) and so 
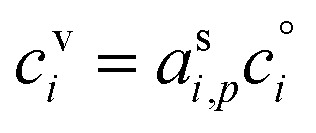
. Fig. 7 shows the equilibrium distribution, with particle composition dominated by the four LVOC constituents. Because the system is at equilibrium but there is no constraint on the particle composition other than ∑*w*_*i*_ = 1, the constituents can appear essentially anywhere on this plot, provided they keep to the appropriate diagonal stripe indicating the Raoult's law equilibrium. The main constraint is that the very low volatility species have very low vapor concentrations. There is no growth-rate axis (and no infeasible region) because there is no growth.

The symbols are filled with a color indicating volatility, which is identical to the color of the underlying stripe; this is proper color order. For these examples, the species have saturation concentrations exactly in the middle of the range defining each bin, and so the symbols appear exactly in the middle of the colored bands; real species will appear anywhere within a given band based on their exact volatility.

#### 7.1.2 Non-reactive particle growth

**Fig. 8:**
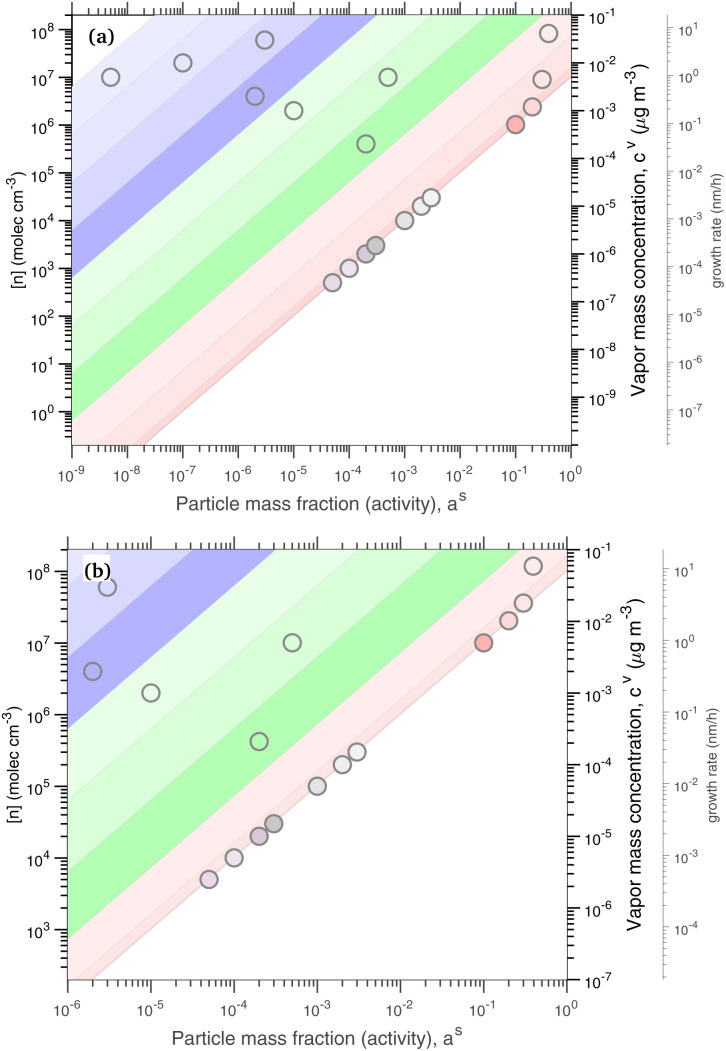
A dVBS showing volatility driven condensation for two growth rates. Vapor concentrations are tied to particle activity (mass fraction) for compounds with saturation concentrations less than the excess vapor concentration, which in turn defines the growth rate. (a) Slow growth rate near 2 nm h^−1^ with an excess vapor concentration of 0.01 μg m^−3^, found at the *a*^s^ = 1 intercept. (b) Fast growth rate near 20 nm h^−1^ with an excess vapor concentration of 0.1 μg m^−3^.

As before, condensational growth implies a steady-state vapor concentration well above the equilibrium, especially for low-volatility species. All the steady-state examples are for continuous growth with steady composition. Fig. 8a shows the distribution for a relatively slow growth rate near 2 nm h^−1^ (*c*^xs^_*p*_ = 0.01 μg m^−3^) and Fig. 8b shows the distribution for growth near 20 nm h^−1^ (*c*^xs^_*p*_ = 0.1 μg m^−3^). The infeasible range is shown in white and appears as an increasing white region advancing from the lower right (high activity, low vapor concentration) toward the upper left (low activity, high vapor concentration) as growth rate and *c*^xs^_*p*_ increases. The colored xLVOC stripes in the log space from the infeasible range collect in a bunch along the diagonal line defined by the growth rate, and the low volatility species collect along that leading edge.

For equilibrium conditions, the various example species appear within their equilibrium VBS ranges; however, for steady-state growth conditions, the low volatility species (with 
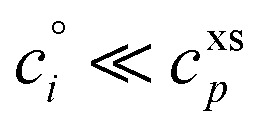
 and thus *γ*_*i*,*p*_ ≃ 1) collect along the limiting line at much higher vapor concentrations. The more volatile species (with 
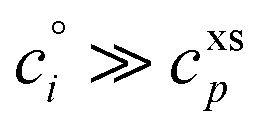
) however still appear near their equilibrium locations. This reflects the qualitative behavior of “quasi irreversible” *versus* “semi-volatile” condensation. Quasi irreversible condensation occurs when *a*^v^_*i*_ ≫ 1 and species line up along the minimum vapor line (or their condensed phase activities are proportional to their relative gas phase concentrations). The colored symbols remain in color order, because the ELVOC and ULVOC diagonal bands are all along the growth-rate diagonal. Semi-volatile condensation on the other hand simply sees the condensed-phase activity remain equilibrated with the gas-phase activity. These colored symbols remain obviously in color order within their diagonal bands. The total semi-volatile activity thus defines a multiplier of growth being driven by quasi-irreversible condensation. This also applies to water vapor; ultimately, if half the volume (mass) fraction of the particles consists of semi-volatile species, then the growth rate is twice the growth rate that would otherwise be from quasi irreversible condensation.

There is almost no middle ground. Vapors are either quasi irreversible or semi-volatile, with only a VBS bin or two with 
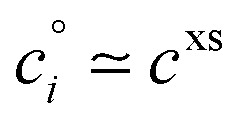
 in transition. This is the basis for the finding that ambient particle growth can be described by a fraction that condenses to particle surface area (implying quasi irreversible behavior) and a fraction that condenses to particle volume (implying equilibration).^48^

In these examples the vapor concentrations do not remain the same, because the condensed-phase activity and the growth rate are specified and the vapor concentrations emerge from those constraints. This is most dramatic for the extremely low volatility species, which are of course almost absent from the vapor at equilibrium but have progressively higher concentrations for progressively higher growth rates. In Fig. 8, the slow-growth case has several LVOC species still above the limiting line, but the fast growth case has largely brought those species in line (with higher gas phase concentrations, along with the other ULVOC and ELVOC species, to collectively drive the faster growth).

#### 7.1.3 Particle growth with inhibited condensation

**Fig. 9:**
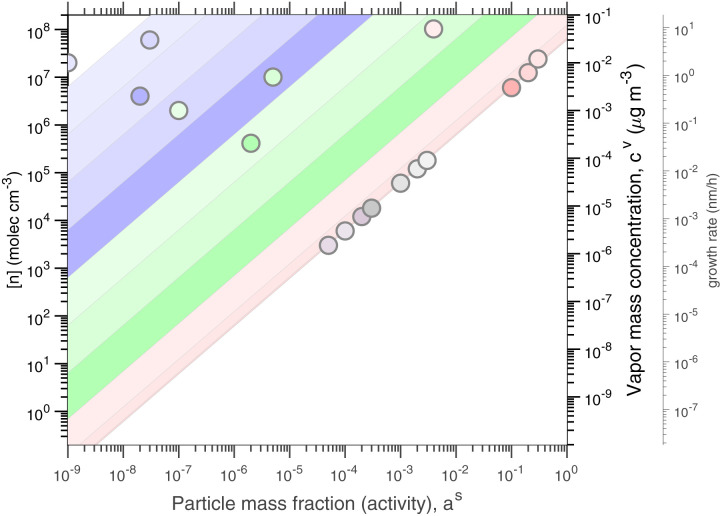
A fast 20 nm h^−1^ dVBS with inhibited uptake of semi-volatile vapors. Condensation of semi-volatile species is inhibited by a factor of 100, dropping their activity and slowing growth. As a consequence, all the semi-volatile species substantially away from the limit line are “out of color order” and displaced leftward to lower (bulk) particle activity.

If (glassy) particles have diffusion limitations to organic uptake due to high viscosity, the surface activity of condensing vapors will remain higher than at steady state and this may inhibit condensation. This will have minimal influence on the effectively non-volatile species with *a*^v^_*i*_ ≫ 1, as *a*^s^_*i*_ ≤ 1 because they will condense onto any surface. However, for species with *a*^v^_*i*_ ≲ 1 the surface activity may come into steady state with the vapor activity and slow condensation (which will be rate limited by diffusion into the particle).^49^ As a consequence, *γ*_*i*_ < *γ*^cond^_*i*,*p*_, and the species will have a lower (bulk) fraction in the particle than expected. Fig. 9 shows an example using the same representative vapor distribution but with semi-volatile condensation inhibited by a factor of 100. The effectively semi-volatile species are displaced to the left of the condensation limit in Fig. 8 by an additional factor of 100. This includes the abundant LVOC species with log_10_ *c*° = 0.1 μg m^−3^, now appearing as a light salmon symbol in a green diagonal, which in the steady-state case comprised 40% of the particle composition but now comprises 0.4% of the particle composition. The growth rate is thus slowed by 40%.

This appearance of lower than expected particle activity – shifting to the left in the particle-vapor phase space – is similar to the signature of reactive uptake, except there is no corresponding product in the infeasible region. Thus in practice it may be difficult to distinguish these two unless they are major contributors to growth (which is fortunately when it is worth telling them apart); the added constraints of slower or faster than expected growth will be an important additional constraint.

The two diagnostic features of inhibited uptake by glassy particles are smaller than expected (bulk) particle phase activity (underrepresentation in the particles) for the same steady-state vapor concentrations as well as (possibly) slowed particle growth if the semi-volatile species comprise a substantial fraction of the condensing vapors and thus have a significant particle-phase activity. As described above, the semi-volatile constituents (including water), with a total activity *a*_sv_, serve to amplify the growth rate by 1/*a*_sv_. Inhibited condensation will dampen this amplification.

#### 7.1.4 Particle growth with irreversible reactive uptake

The “speed limit” on condensational growth is the collision rate of vapors with particles (*γ* = 1), but if a relatively volatile species (such as the IVOC in our example mixture) reacts in the condensed phase to form a much less volatile product, *γ*_*i*_ ≫ *γ*^cs^_*i*_, and otherwise slow condensation will be pushed up to that speed limit. For these limiting cases we are only considering those where the vapor concentrations and condensed phase activity remain constant, but that is sufficient to illustrate the characteristics of reactive uptake. The simplest case to consider is a semi-volatile reactive species that only reacts with itself, rate limited by condensation with *γ*_*i*_ ≃ 1. This will not change the concentration of the condensing vapor in this example, but it will change the condensed phase activity of that species in the particle.

**Fig. 10:**
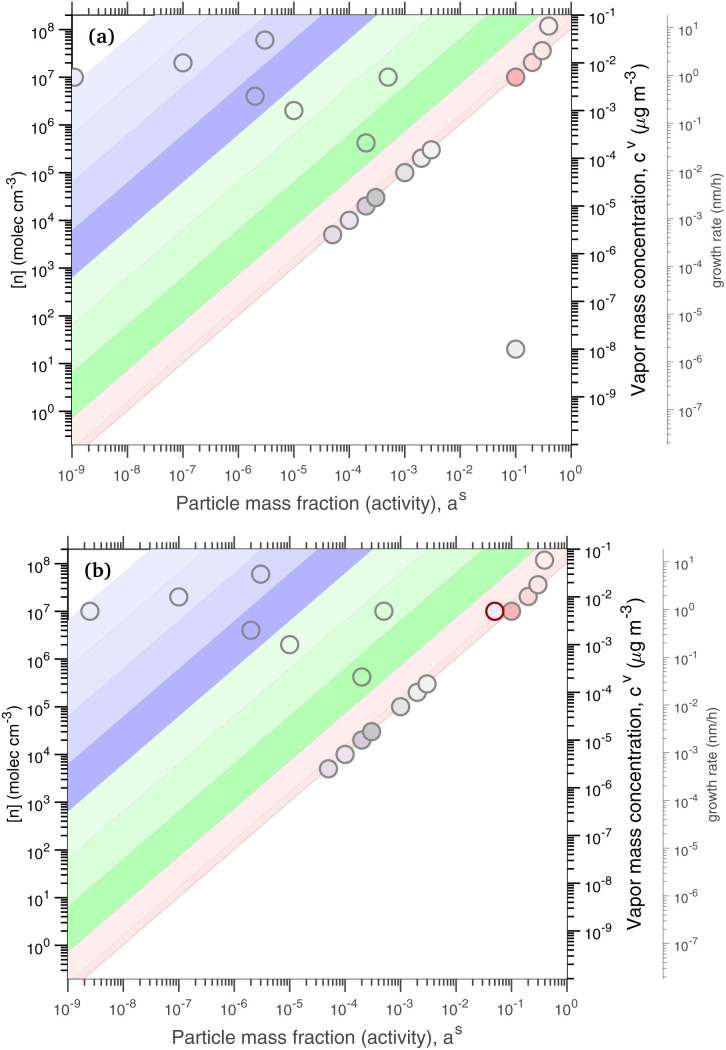
Fast 20 nm h^−1^ dVBS with reactive uptake of an IVOC monomer (with light blue color at the far left). (a) The product is a thermally stable ELVOC dimer. Rapid dimerization drops the IVOC activity (*a*^s^) to near zero (blue circle to far left at 10^7^ cm^−3^) and yields a dimer in the infeasible region (with very low vapor concentration and *a*^s^ = 0.1). Both species appear out of color order but the stable ELVOC stands out in the infeasible region (b) the product dimer thermally dissociates when being measured by temperature programmed desorption. Now the monomer appears near the limiting diagonal, still at 10^7^ cm^−3^ (with a red-edged symbol indicating thermal decomposition filled with light blue indicating IVOC monomer volatility).

Fig. 10a shows this simple example for the most volatile IVOC in the mixture (colored light blue at the far left). In this case the activity of the volatile monomer drops to near zero in the condensed phase and the activity of the product rises to the maximum given by unit uptake. Both the monomer and the dimer appear in regions of the phase space inconsistent with non-reactive condensation (they are out of color order); the monomer is depleted in the condensed phase and the dimer appears in the infeasible region for condensation. Within the mixture, the monomer displacement (toward the *y*-axis) is relatively subtle (it is shifted from the center of the left-most blue diagonal), but the product dimer stands out in the infeasible range.

#### 7.1.5 Particle growth with reversible reactive uptake

The thermally reversible uptake covered above is depicted in Fig. 10b for the same IVOC monomer and the same overall (mass) activity with uptake coefficient, *γ*_*i*_ = 0.5. Here, rather than just having an unusually low condensed phase activity, the monomer appears twice, once with low but once with unusually high condensed phase activity. Here the signal appears among the other species undergoing quasi-irreversible condensation and so the major indication is that the compound should otherwise be in the quasi-equilibrium region, so it is out of color order. The TPRS thermogram will also have two peaks, one appearing at the proper temperature associated with the monomer volatility, and a second at a much higher temperature (defined not by the dimer volatility but rather by the decomposition temperature of the dimer). In this example the first TPRS peak would be extremely small (the particle mass fraction of the pure monomer is *a*^s^ = 3 × 10^−9^) but the second peak would be large (*a*^s^ = 0.045). The dVBS color-order discrepancy may be difficult to discern in real-world data, but the multiple TPRS peaks with uncharacteristic appearance temperatures will be readily evident.

### 7.2 Specified vapor composition

**Fig. 11:**
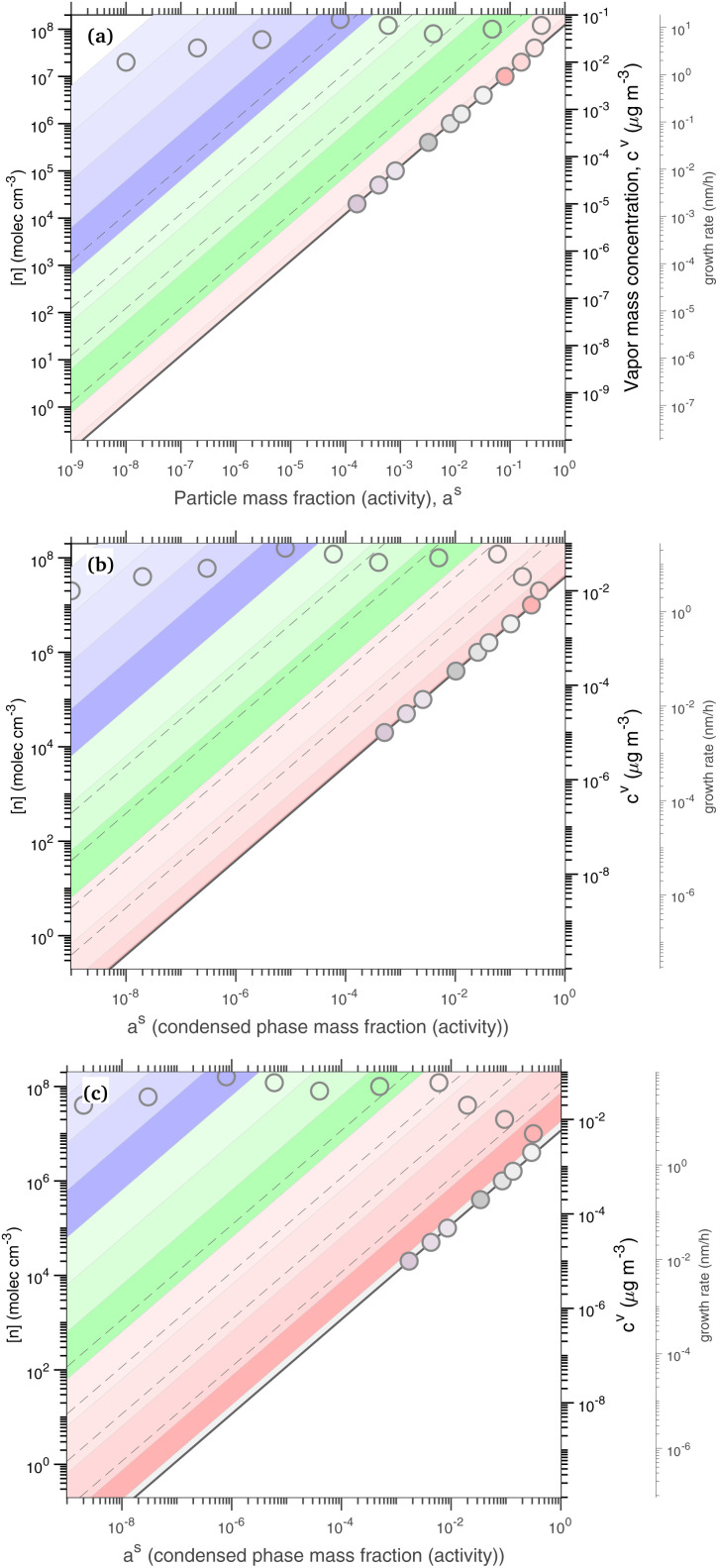
Non reactive condensation with a single specified gas-phase concentration ensemble for a progression of particle sizes (Kelvin terms and microphysical growth enhancements). The simple dVBS nomenclature is insufficient. (a) Large particles with *K* = 1 giving a simple 10 nm h^−1^ dVBS with *c*^xs^ = 0.06 μg m^−3^. (b) Particles near the Kelvin diameter with *K* = 10. This is a 10 K 10 nm h^−1^ dVBS with *c*^xs^ = 0.012 μg m^−3^ and an actual growth rate of 4 nm h^−1^. (c) Tiny particles with *K* = 100. This is a 100 K 10 nm h^−1^ dVBS with *c*^xs^ = 0.006 μg m^−3^ and an actual growth rate of 5 nm h^−1^.

Instead of specifying the particle composition we can specify vapor concentrations for non-reactive condensation, and then solve iteratively for the particle composition under the constraint that ∑*w*_*i*_ = 1. As an example we specify a distribution with IVOC through SVOC species all near 10^8^ cm^−3^ (simply to keep the plot in range with a tail of progressively lower concentrations through the LVOC, ELVOC, and ULVOC ranges). Now the vapors determine the growth rate and composition. Fig. 11a shows this example case, which results in a growth rate near 10 nm h^−1^ and an excess concentration, *c*^xs^ ≃ 6 × 10^−2^ μg m^−3^. This is a 10 nm per h dVBS, for relatively large particles, where *K*_*i*,*p*_ ≃ 1 and the deposition speed is simply *s*/4. The solid diagonal establishes the growth rate and defines the infeasible region. The four dashed diagonals displaced from the growth-rate diagonal show log_10_ *γ*_*i*_ = −1, − 2, − 3, and −4.

For smaller particles, the Kelvin term becomes significant and the condensation speed is enhanced by a combination of the reduced mass, finite molecular size, and van der Waals terms. Fig. 11b shows a case with the same vapor concentrations for *K* = 10 and Fig. 11c shows a case with *K* = 100, corresponding to *d*_*p*_ of roughly 4 and 2 nm. The dVBS nomenclature includes the Kelvin term, so these are a *K*10 and *K*100 10 per nm dVBS. The particle curvature (Kelvin term) raises the effective activity and so various shades of LVOC emerge from the quasi-irreversible limit line for smaller particles; however, with the fixed vapor concentrations, this also lowers the excess saturation ratio and enriches the particles in the less volatile constituents, which move to the right in the figure. Thermodynamics determine the excess concentration and are independent of the growth-rate axis, which in turn shifts downward as the growth enhancement terms increase.

During actual particle growth, even for constant vapor concentration, the system will sweep through these conditions and so a truly constant activity steady-state solution is not exact. The less volatile species are favored and enriched in small particles because of the Kelvin effect, and this demonstrably slows growth in the early stages because the relatively more volatile species (LVOCs) do not condense on the smallest particles.^20,22,26^ In many cases those ULVOCs and ELVOCs that condense first are also covalently bound dimers,^50^ and so may also be more likely to undergo thermally reversible decomposition. Overall, while there will be some residual enrichment in larger particles, because of the *d*_*p*_^3^ volume dependence this should be modest for particles larger than 10 nm or so; a full microphysical simulation (not shown) confirms that the enrichment is almost always quite modest.

This dVBS methodology is suitable for near steady-state conditions with a known growth rate, especially to diagnose well constrained experiments and to test for closure between measured vapor concentrations, growth rates, and particle composition. For ambient measurements away from steady-state conditions, species could appear in the infeasible region simply by having been deposited by condensation by a vapor no longer present. However, because volume scales with the cube of diameter, such a cutoff would need to be recent for the mass fraction to be large. The dVBS will thus also be useful for analysis of real-world data, provided that the particle history is sufficiently well constrained. It will always reveal what the relationship would be between particles and vapors for a given growth rate, if the system were at steady state for its entire history.

## 8 CLOUD observations

To test the predictions of particle composition, we turn to data from the Cosmics Leaving Outdoor Droplets (CLOUD) experiment at CERN.^51,52^ Specifically, we measured the composition of particles formed following α-pinene ozonolysis during the Autumn 2019 CLOUD 14 campaign; these were “pure biogenic nucleation” runs. During these runs, CLOUD was maintained at *T* = 243 K and variable RH. The experimental conditions are described in detail in Surdu *et al.*, 2023.^53^

We need four things to fully compare gas and particle-phase abundance and composition and to interpret the data with this diagonal Volatility Basis Set. First, we need to know the relevant growth rates and thus excess concentration. Second, we need accurate measurements of gas-phase concentration. Third, we need precise measurements of the (total) particle-phase composition (mass fractions). Fourth, we need to know the volatility of the measured molecules. The volatility in the dVBS is always the volatility at the given temperature, so all volatility values and all colored symbols and bands are for *c*°(243).

### 8.1 Particle size and growth rates

**Fig. 12:**
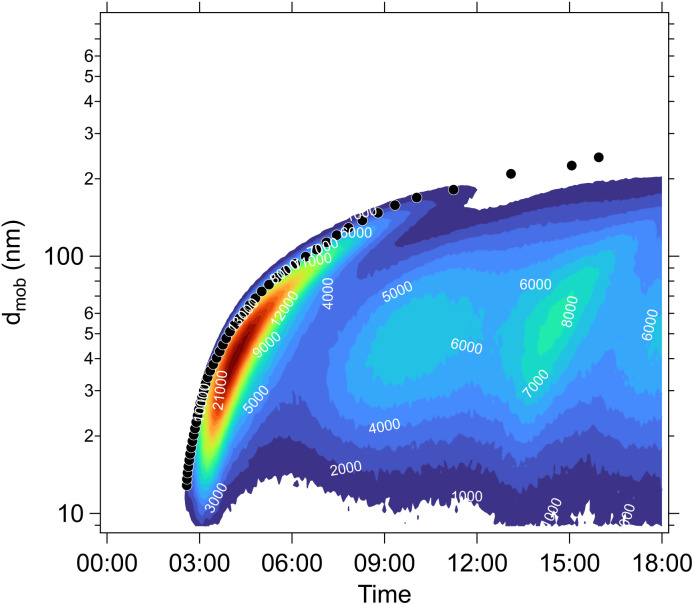
Particle size distribution during an α-pinene ozonolysis nucleation and growth run at 243 K during CLOUD 14 at CERN in fall 2019. Contours show the magnitude of the size distribution (d*N*/dlog *d*_*p*_). Black circles show the leading edge of the size distribution, indicating a particle growth rate between 15 and 30 nm h^−1^.

Fig. 12 shows the evolving particle size distribution measured with a Scanning Mobility Particle Sizer (SMPS) during run 2210, with the leading edge marked with circles. The growth rate was initially near 30 nm h^−1^ and gradually slowed to 15 nm h^−1^. Two secondary nucleation modes formed as the particles grew and deposited to the chamber walls, dropping the condensation sink sufficiently to allow the nucleating vapors (likely ULVOC “dimers”^54–56^) to initiate secondary nucleation bursts. These show qualitatively similar growth rates. For the purposes of this assessment, the growth rate between 30 nm h^−1^ slowing gradually to 15 nm h^−1^ is close enough to constant; this means that the total excess concentration was *c*^xs^ ≃ 0.1 μg m^−3^. We therefore expect species with *c*°(243 K) ≲ 0.1 μg m^−3^ to collect along a “condensation line”, with more volatile species disproportionately favoring the gas phase with respect to that line.

### 8.2 Gas and particle measurements

Here we use I^−^ chemical ionization mass spectrometer measurements by a Filter for Gases and Aerosols (FIGAERO) instrument.^46,57,58^ Measurements alternated between direct gas-phase sampling from the chamber and temperature programmed desorption of particles collected on a Teflon filter during the preceding gas-phase measurement interval. We are not confident in the absolute calibration of FIGAERO signals during this campaign, in part because transmission of the primary reagent ion (I^−^) through the time of flight mass filter was low. Consequently, here we restrict ourselves to interpreting the raw signals. A full closure analysis using the dVBS is in a companion publication describing particle growth from isoprene oxidation products at low temperature.^59^ We thus implicitly assume that the sensitivity of the I^−^ CIMS is the same (on average) for all of the measured species, and to obtain signals proportional to the mass concentration we multiply the raw signals by the atomic mass of the measured species. Provided the sensitivity of the I^−^ CIMS is reasonably constant, and that we measure all the major constituents of the particles, the FIGAERO should yield precise overall mass fractions. The bulk of the species measured here are highly functionalized C_10_ and C_20_ compounds that are likely near the maximum sensitivity for I^−^ collisions, but in general the FIGAERO sensitivity will vary depending on the cluster binding energy.^60^

### 8.3 Estimated and measured volatility

The FIGAERO thermograms provide a direct measurement of volatility based on appearance temperature^58^ and can also reveal the presence of products that thermally decompose during desorption – these may be products formed *via* condensed phase chemistry.^47^ Past work has shown excellent correspondence between the measured volatility^57,58^ and a composition-activity relation specific to these products that have undergone a high degree of autoxidation, described in Stolzenburg *et al.*^26^ For this campaign the position of the thermocouple measuring heated carrier flow temperature was uncertain, and calibrations were not obtained. We therefore use the well established composition-activity measurement as our primary measure of volatility and use a best fit for compounds with a single well-defined desorption peak and *n*_C_ > 12 to relate peak desorption temperatures to volatility, as described in the ESI.†

#### 8.3.1 Thermogram classification

To assess the behavior of this system, we group the species observed in the FIGAERO thermograms into four categories:

(1) Species with a single thermogram peak and *n*_C_ ≥ 8.

(2) Species with *n*_C_ ≤ 7.

(3) First peak in thermograms with multiple peaks and *n*_C_ ≥ 8.

(4) Later peaks in thermograms with multiple peaks and *n*_C_ ≥ 8.

**Fig. 13:**
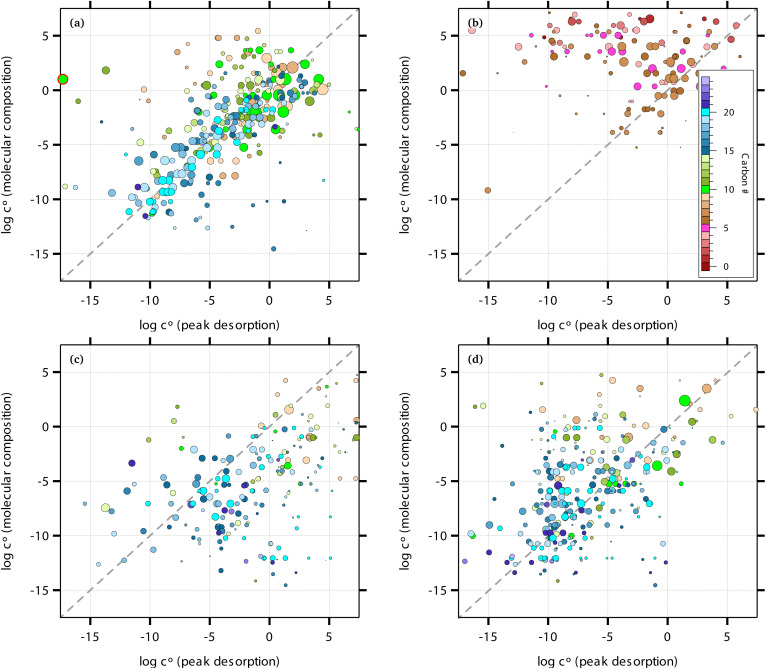
Compounds in different thermogram categories near 15:00 UTC (temperature ramp 25) during Run 2210. Symbol colors show the carbon number, *n*_C_, and symbol sizes are proportional to (the log of) the thermogram peak area. (a) Compounds with a single thermogram peak and *n*_C_ ≥ 8. The large majority fall near the empirical 1 : 1 line shown as a diagonal in the figure. A prominent C_10_ peak with unusually high desorption temperature (low volatility) but much higher volatility based on composition is shown with a red-edged circle. (b) Compounds with *n*_C_ ≤ 7. These are almost certainly thermal decomposition fragments. Their apparent volatility (based on appearance temperature) is uncorrelated with their uniformly high volatility based on composition. (c) Compounds with *n*_C_ ≥ 8 that appear first in a thermogram with multiple peaks. These may be isomers with unusually high volatility compared to the nominal composition-activity relation. (d) Compounds with *n*_C_ ≥ 8 that appear later in a thermogram with multiple peaks. These may include some isomers but also thermal decomposition products.

Fig. 13 shows the compounds observed in each of these categories. The symbol sizes are proportional to the log of the peak area for each peak in the thermogram. The symbol colors in this figure show carbon number, *n*_C_, and not volatility as in the dVBS figures, and so the color scale is different. Bright colors for *n*_C_ = 5, 10, 15, 20 show values most expected from terpene (C_10_) chemistry.

Fig. 13a shows the larger (*n*_C_ ≥ 8) compounds with only one thermogram peak. They comprise the very large majority of the total condensed phase signal. These mostly fall near the 1 : 1 line and are consistent with robust monomers and dimers desorbing as such from the filter at a temperature consistent with their nominal volatility. We therefore conclude that most of the compounds forming these particles are more or less standard oxidation products of α-pinene. However, at this point in the analysis it is not yet possible to determine whether the dimers formed in the gas phase and then condensed, or whether they were formed by condensed-phase association reactions after monomer condensation; that requires a dVBS analysis. A prominent C_10_ peak falls well off the diagonal; this is identified with a red border.

Fig. 13b shows the small (*n*_C_ ≤ 7) species. These are consistent with thermal decomposition products not comprising simple reversal of a dimer reconstituting a monomer. They comprise roughly 15% of the total (mass weighted) condensed-phase signal and roughly half of that is well away from the 1 : 1 diagonal. Their appearance temperature (and thus apparent volatility) is driven by their decomposition temperature and not their volatility, and so there is no correlation between their composition and apparent volatility. There is no reason to expect such a correlation; especially for highly functionalized molecules, there may be many reaction pathways involving fragmentation into two smaller molecules, and provided that the activation energy for that decomposition reaction is lower than the desorption enthalpy for the parent molecule, the fragments will appear before the parent desorbs. This is well established for highly oxygenated organic aerosol^61^ and relatively weakly bound oligomers.^47^ Provided that both products are sufficiently volatile, they should appear in pairs,^47^ but for data as complex as ours it is impossible to discern these pairs. Roughly half the small molecules do appear near the 1 : 1 line, consistent with simple desorption, and while a small portion with *n*_C_ = 6, 7 arguably are misclassified (to the extent this figure targets decomposition fragments), for most it is impossible to distinguish decomposition and simple desorption.

Fig. 13c shows the first peaks for compounds with multiple peaks in a thermogram. These include some peaks with apparently high volatility, as expected because they have the lowest appearance temperature of a group of peaks with the same composition. Overall they form three groups. One falls close to the nominal 1 : 1 line; this is consistent with monomers showing nominal volatility that either have companion isomers with unusually low volatility or are monomers present as free species in the particles but also formed by the thermal decomposition of larger compounds in the particles. The second group consists of several clusters of species with unusually high volatility. It is interesting that these are largely absent from the compounds that show only a single thermal desorption peak in Fig. 13a; these may be isomers of other species that have unusually high volatility due to a preponderance of oxygen atoms appearing in the carbon backbone (*i.e.* ROR and ROOR functional groups), which have a modest effect on volatility.^62^ The third group consists of peaks with low apparent volatility, even though they are the first in a set of multiple peaks.

Fig. 13d shows the later peaks for compounds with multiple peaks in a thermogram; it is thus the complement to Fig. 13c. Again, many of these peaks (most of the signal) fall along the 1 : 1 line and so are consistent with “well behaved” compounds with nominal volatility; these are likely more typical isomers paired with the unusually volatile isomers in Fig. 13c. Some peaks, especially “dimers” with *n*_C_ ≃ 20 may be isomers with unusually low volatility compared with the composition activity relation (for example isomers with more –OH functional groups than the typical products. There is a hint of a horizontal band of species with 8 ≤ *n*_C_ ≤ 10, which could be monomeric decomposition products of larger association products (“dimers”); however, these represent a small fraction of the total mass compared to the C_20_ dimers.

#### 8.3.2 Overall volatility behavior

**Fig. 14:**
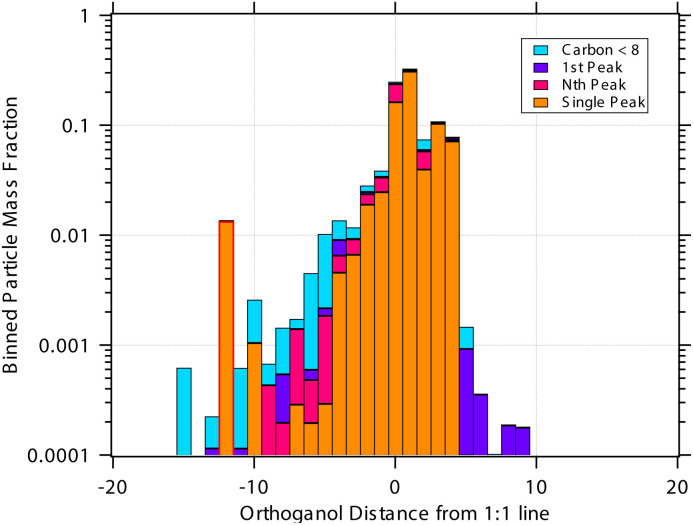
Histogram of particle mass fraction binned by distance (in log_10_ units) from the 1 : 1 line for perfect correspondence between apparent volatility and volatility based on the composition activity relation in Stolzenburg *et al.*^26^ Most of the signal resides in compounds with a single peak in the temperature programmed desorption thermogram, shown in orange; roughly two thirds of the signal falls within one bin (one order of magnitude) of this line.

Fig. 14 shows the mass distribution as a histogram based on distance (in decades) from the 1 : 1 line for calculated *vs.* apparent volatility. On average the agreement is excellent, but this is expected as we used the calculated volatility of larger molecules with well defined single peaks to establish our correlation. However, most of the overall mass was not used for that empirical fit and still agrees well. Ultimately, more than two-thirds of the total mass falls within about a decade of the expected volatility for “well behaved” molecules that condense and desorb from particles based on their volatility. Further, the histogram is color coded as a stack plot according to the four classifications just discussed. The large majority of the mass is found in peaks that are not just well behaved but show a single thermogram peak.

The red-edged orange bar in Fig. 14 with an orthogonal distance of −12 is almost entirely the C_10_ compound with a red-edged symbol in Fig. 13a, which overall comprises just over 1% of the particle signal. A second bar at −10 bar is an (unusual) C_11_ product that also appears in Fig. 13a. While noteworthy, these compounds comprise a small fraction of the total mass.

The (low temperature) composition (and growth) of these particles is almost entirely dominated by a few monomer products, even though the nucleation itself was rate-limited by ULVOC dimers.^54,56^ These six most abundant species comprise 50% of the total mass signal: C_10_H_16_O_5_ (11%); C_8_H_12_O_4_ (10%, C_8_ diacid or isomer)); C_10_H_16_O_4_ (10%, hydroxypinonic acid or isomer); C_9_H_14_O_5_ (6.5%); C_10_H_16_O_6_ (6.5%); and C_10_H_16_O_3_ (6%, pinonic acid or isomer). Some of these correspond to well known major products of α-pinene ozonolysis,^63,64^ with some O_5_ and O_6_ species consistent with autoxidation and formation of highly oxygenated organic molecules (HOMs).^65,66^

### 8.4 Comparison of gas and particle phase signals

As it appears that most of the (mass) signal in the particles consists of “well behaved” molecules that desorb with a volatility consistent with the Stolzenburg composition activity relation,^26^ we can then explore whether the observed vapor and particle signals behave as expected in the “diagonal” VBS. We know the total excess concentration is roughly 0.1 μg m^−3^, but we are not confident in the gas-phase absolute calibration and so cannot directly assess full closure of the dVBS. However, we do expect species with *c*° ≪ 0.1 μg m^−3^ to fall within an order of magnitude or so of a 1 : 1 condensation line, and those with *c*° ≫ 0.1 μg m^−3^ to fall above (to the upper left) of that line, with more volatile species lying farther from the line. This is “color order” in the dVBS.

**Fig. 15:**
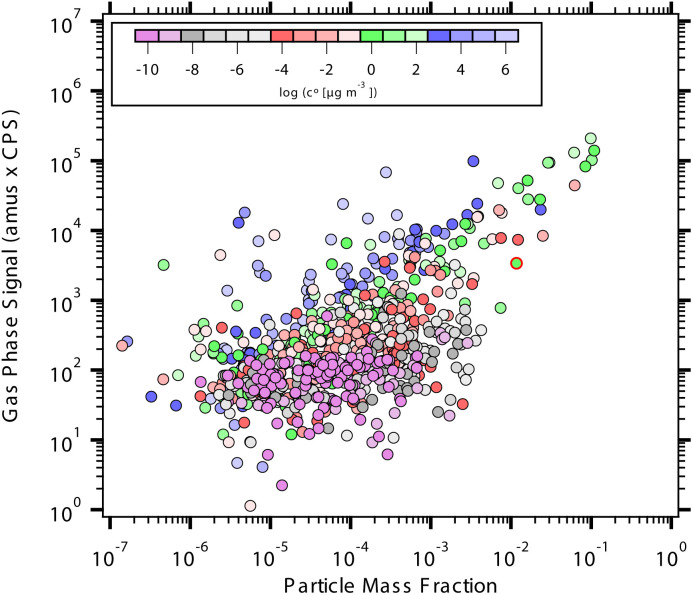
A dVBS for species measured with I^−^ FIGAERO near the end of the biogenic nucleation run. Both signals are weighted by the molar mass of each peak and the symbol color indicates the volatility bin (log *c*°) given by the color scale. Volatility is determined by the composition of each peak. Gas and particle signals are generally correlated, with more volatile species having relatively higher gas-phase signals. There is no obvious signal in the infeasible region toward the lower right, where a species formed *via* condensed-phase chemistry would have high particle-phase signal but low corresponding gas-phase signal.

Fig. 15 shows the observed gas and particle phase signals near the end of this run. We multiplied the raw instrument signals (cps) by the molecular weight of each species to estimate the mass concentration and mass fraction. As with all dVBS plots, symbol colors now indicate saturation concentration (log_10_ *c*°) at 243 K, shown with the horizontal color bar, and size is constant as the mass fraction is now the *x*-value itself. Signals in the two phases are strongly correlated, and the observations also clearly fall in color order with the more volatile species exhibiting higher gas-phase signals at a given particle mass (signal) fraction. The lowest signals tend to collect between 10–100 arbitrary units on the *y*-axis and *w* < 0.001 on the *x*-axis; this is near the detection limits of the gas and particle measurements.

In Fig. 15, the purple, gray and some salmon symbols (ULVOC, ELVOC, and some LVOC) fall in a group along the lower edge of the data, while the some of the salmon and most of the green and blue (LVOC, SVOC, and IVOC) symbols fall above this group by a factor of roughly 10–100, with the IVOC species falling above the SVOC species. For particles growing at 15–30 nm h^−1^, the color arrangement evident in Fig. 15 is consistent with our theoretical expectations illustrated in Fig. 2. For non-reactive uptake, all the ULVOC and ELVOC and some of the LVOC should fall along the condensation limit line, and the more volatile species should fall above that line.

We lack the gas-phase calibration to situate the condensation line accurately in Fig. 15, but for 20 nm h^−1^ growth shown in Fig. 12 the total excess concentration is *c*^xs^ ≃ 0.1 μg m^−3^. Extrapolating the red (LVOC) symbols by eye, the (right-hand) *y*-intercept of the condensation line should thus be near 3 × 10^5^ arbitrary units of the *y*-axis in Fig. 15. This in turn would be roughly 2 × 10^8^ cm^−3^, meaning the vapor concentrations span a range from 10^8^ cm^−3^ to 10^5^ cm^−3^ before reaching the evident detection limit near 100 arbitrary units in the figure. This is broadly consistent with the expected vapor concentrations^55,67^ and overall FIGAERO I^−^ sensitivity.^60^ We again identify with a red border the C_10_ monomer that is an obvious thermal decomposition product in Fig. 13a and 14. Intriguingly, this falls near where we expect the diagonal condensation line would be with a sufficient gas-phase calibration. This is thus consistent with reactive uptake of this species, followed by thermal decomposition. Falling near or on the condensation line, it would have a high uptake coefficient and consequently the monomer itself would be depleted in the particles, as illustrated in Fig. 6a. For this reason, there would only be a single peak in the thermogram, as we observe.

The most notable feature of Fig. 15 is that there are no obvious peaks with high particle mass fractions and low gas-phase signal. The infeasible region illustrated in Fig. 4 that can be populated only by reactive uptake to form low-volatility products is essentially empty. This is consistent with the thermogram analysis and the high proportion of single thermogram peaks that fall near the expected volatility line in Fig. 13a. Along with the obvious C_10_ thermal decomposition product, there are other peaks consistent with reversible reactive uptake. By combining the dVBS and thermogram analysis, this appears to comprise no more than 10% of the total particle mass.

There are few species in Fig. 15 displaced dramatically to the left, especially with high vapor signals. Just as the composition analysis in Fig. 13 shows that most of the peaks and most of the signal consists of relatively well behaved compounds, with a small amount of evident fragmentation, this dVBS is qualitatively consistent with our expectations. Thus these data appear to be consistent with volatility limited condensation being responsible for the large majority of particle growth, augmented with a few percent of thermally reversible reactive uptake.

### 8.5 Observation summary

Overall, the combined gas and particle measurements with the I^−^ FIGAERO instrument are consistent with particle growth being dominated by condensation of species formed *via* gas-phase oxidation chemistry of α-pinene. There is no sign of a large contribution to the overall particle mass from formation of a unique species *via* condensed phase chemistry (that would appear in the infeasible region of Fig. 15). There is also no obvious sign of inhibited uptake of semi-volatile species associated with low relative humidity and glassy particles early in the run. Most of the TPD peaks fall reasonably in line with their expected volatility, as shown in Fig. S2,† but some signal is clearly associated with decomposition into C_10_ and C_5_ fragments.

The absolute calibration of especially the gas-phase signals was not sufficient to test the quantitative constraint of *c*^xs^ (the *y*-intercept) *vs.* observed growth rate. Further, the I^−^ CIMS certainly does not measure all gas-phase species and likely misses some condensed-phase material as well.^68^ If the unobserved condensed-phase species contribute substantially to the particle mass, that would also affect the calculated mass fractions of all compounds. Thus, while we can conclude that these observations are consistent with the theoretical expectations of growth driven principally by condensation based on volatility, complete and quantitative closure requires thorough calibration and likely constraints from multiple instruments.

## 9 Conclusions

The diagonal Volatility Basis Set establishes a quantitative phase space that combines particle microphysics with the equilibrium thermodynamics of VBS phase partitioning for organic mixtures to diagnose the processes governing growth. Steady-state growth of particles requires a certain excess concentration of condensible vapors, and for uptake of low volatility species (LVOC and below), the only reason they will be found in the gas phase at any significant concentration is because there is significant gas-phase production balanced by condensation to particles. Simultaneous measurements of vapor concentrations and (complete) particle phase mass fractions, along with measured particle growth rates, permits a constrained test of the observations for closure. It is notable that the requirements for the gas phase (absolute accuracy of concentration measurements) differ from those for the particle phase (accurate determination of composition – mass fraction – for all species).

The dVBS space defines an infeasible region where species with high particle mass fractions but lacking a corresponding high vapor concentration can only have been formed *via* condensed phase chemistry. Depending on the volatility of the associated vapor precursors, this will also reveal whether the condensed-phase chemistry is rate limiting for particle growth. Data on α-pinene + O_3_ products at 243 K from the CLOUD chamber suggest that condensed phase chemistry is not significant for this system.

## Abbreviations

Terms and indexing used in this work. For the general definitions of subscripts and superscripts, we use the square symbol and underscores to represent “any”, with the term in the indicated position being defined. For example, □^_,t^__,__ indicates the superscript “t” in the second position for any term.

□^v,_^__,__: in (from) vapor phase
□^_,b^__,__: in (to) suspended particle bulk
□^_,s^__,__: in (to) suspended particle phase
□^_,u^__,__: in (to) suspended particle surface
□^_,_^_*i*,__: of species *i*
□^_,_^__,*p*_: in (of) particle population *p*
□^_,_^_*i*_: of species *i*, over all particle populations
□^_,_^_*p*_: of all species in particle population *p*
□_,_: all species in all particle populations
□°: (pure) saturation
□^⊥^: perpendicular (to particle surface)
□^cs^: condensation sink
□^gr^: (particle) growth
□^xs^: excess
□^e^: effective
*a*
: activity
*B*
: gas-phase diffusion limitation
*c*
: concentration
*d*
: diameter
*E*_μ_
: electrostatic enhancement factor
*e*
: reduced mass correction factor
K: Kelvin (curvature) enhancement
*k*
: collision coefficient
*m*
: mass
*N*
: number concentration
*S*
: saturation ratio
*s*
: speed
*w*
: mass fraction
*x*
: mole fraction
*α*
: mass accommodation coefficient
*ε*
: vapor size correction factor
*γ*
: uptake coefficient
*ν*
: specific volume
*Φ*
: total flux (per unit air volume)
*ϕ*
: flux (per unit particle surface area)
*σ*
: cross section
*ς*
: surface tension
*ρ*
: density
*ζ*
: activity coefficient

## Conflicts of interest

The authors declare no conflicts.

## Supplementary Material

EA-005-D5EA00062A-s001

EA-005-D5EA00062A-s002

EA-005-D5EA00062A-s003

## Data Availability

Data for all figures in this paper are available on the CERN Zenodo server.
