# Supplementary material for: A diagonal volatility basis set to assess the condensation of organic vapors onto particles
Source: Environ Sci Atmos. 2025 Jul 21;5(9):1035–61. doi: 10.1039/d5ea00062a (PMC12314873; doi:10.1039/d5ea00062a)
Supplement: EA-005-D5EA00062A-s001 [file EA-005-D5EA00062A-s001.pdf]

Supplementary Information (SI) for Environmental Science: Atmospheres.
This journal is © The Royal Society of Chemistry 2025

Environmental Science: Atmospheres

ROYAL SOCIETY OF CHEMISTRY

SUPPLEMENTARY INFORMATION

A diagonal volatility basis set to assess the condensation of organic vapors onto particles

Brandon Lopez, Nirvan Bhattacharyya, Jenna DeVivo, Mingyi Wang, Lucia Caudillo-Plath, Mihnea Surdu, Federico Bianchi, Zoé Brasseur, Angela Buchholz, Dexian Chen, Jonathan Duplissy, Xu-Cheng He, Victoria Hofbauer, Naser Mahfouz, Vladimir Makhmutov, Ruby Marten, Bernhard Mentler, Maxim Philippov, Meredith Schervish, Dongyu S. Wang, Stefan K. Weber, André Welti, Imad El Haddad, Katrianne Lehtipalo, Markku Kulmala, Douglas Worsnop, Jasper Kirkby, Roy L. Mauldin, Dominik Stolzenburg, Siegfried Schobesberger, Richard Flagan, and Neil M. Donahue

S1 Supplementary Information

The relation between peak desorption temperature and volatility (really ΔH^{vap}) is well established for robust species that do not decompose before evaporating.[1,2]

S1.0.1 thermogram volatility calibration

In general, if a given peak in the FIGAERO I^- CIMS measurement is from a single robust isomer we would expect a one-to-one correspondence between the desorption (appearance) temperature and the true volatility. However, there may be several isomers with different volatilities, and furthermore compounds may fragment either during thermal desorption or as a result of the ion-molecule reaction. Regardless, we expect the composition-activity relation to be the most precise measure of true volatility (as it does not change), whereas the thermogram is subject to noise and other sources of error.

In this dataset, many species in the FIGAERO temperature-programmed desorption thermograms show clear, single peaks, while others have more complicated shapes and multiple peaks. We shall assume that the single thermogram peaks other than those with low carbon number are robust and unique isomers. In Fig. S1 we plot the volatility based on the formula (composition activity relation) for compounds vs the peak desorption temperature, $T_{\max}$, with carbon number shown by the symbol color and (log) signal fraction shown by symbol size. We emphasize notable carbon numbers (given the C_{10} α-pinene precursor, $n_C = 5, 10, 20$) with brighter colors.

Fig. S1 has several interesting features. First, there is a clear inverse relationship between peak desorption temperature and the composition-activity relation, with an obvious group of C_{10} species along the diagonal at low $T_{\max}$ and a distribution of C_{20} species along the diagonal at high $T_{\max}$. One prominent C_{10} species, with relatively high signal, appears at very high $T_{\max}$ – this is almost certainly a dimer decomposition fragment, as discussed in the main text. There are very few species well to the

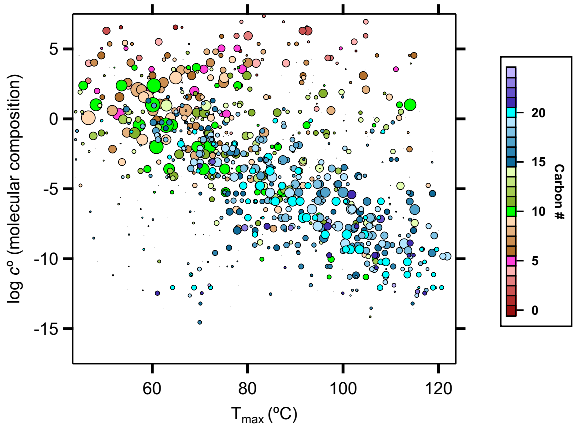

Fig. S1 Volatility calculated using the established composition-activity relation for terpene oxidation products ($\log c^{\circ}$) vs maximum desorption temperature ($T_{\max}$) for thermograms with a single, well behaved desorption peak and $n_C > 12$. We use a linear regression (shown as a red line) to establish the apparent volatility based on observed $T_{\max}$ for all thermogram features.

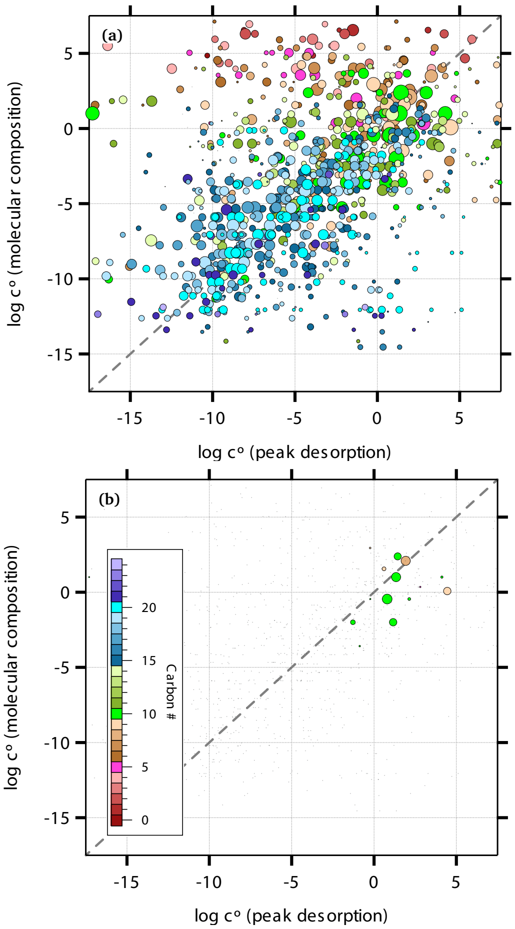

Fig. S2 Calculated volatility ($\log c^\circ$) based on molecular composition vs apparent volatility based on peak desorption temperature. Symbol colors show carbon number (n_C) and symbol sizes are proportional to the (*log* of) peak area. Overall, calculated and measured volatility agree well, but there are evident lobes of peaks, especially for $n_C < 8$ as well as for C_{10}, consistent with some thermal decomposition during the temperature ramp. (a) All species with symbol size proportional to $\log c^\circ$. (b) All species with a compressed range for symbol size, with all peaks smaller than 1% of the largest peak at the minimum size; the 6 most abundant peaks comprise half the total observed particle mass.

lower left of the diagonal. These would be compounds expected to have low volatility that none-the-less appeared at low temperature; it makes sense that these are rare. In contrast, there are many species to the upper right of the diagonal, especially with $n_C \lesssim 7$, but also including the prominent C_{10} outlier. There is no evident correlation between the expected volatility and T_{max}, consistent with thermal decomposition and not volatility being their source. Almost no species in this decomposition group have $n_C > 10$.

All in all, most of the peaks in Fig. S1 are well behaved and fall along the expected diagonal relating desorption temperature and volatility. We thus use a log-linear regression on peaks with a single thermogram peak and $n_C > 12$ to find an empirical calibration of volatility vs T_{max}, labeled "peak desorption", for the remainder of this discussion.

S1.0.2 calculated vs observed volatility

In Fig. S2 we compare the calculated volatility of each species with the volatility based on the peak desorption temperature; the symbol colors show carbon number and the symbol sizes are proportional to the log of the signal. In all these figures we use the same volatility range spanning 20 decades ($-17.5 < \log_{10} c^\circ < 2.5$) and the same color scale for carbon number. The clear correlation along the 1:1 line is expected given the empirical calibration, though this continues for $n_C < 12$, including most of the total signal. Much of the visible range of the figure is spanned by C_{20} "dimers" and the C_{10} monomers along that common 1:1 line. This also confirms that the C_{20} species are covalently bound because clusters would not survive thermal desorption.[1]

However, Fig. S2 also has numerous peaks far from the 1:1 line. Much of the figure is occupied by dimers with $n_C \simeq 20$. Compounds with $n_C \lesssim 7$ have an appearance temperature uncorrelated with their formula base volatility, appearing as a horizontal band along the top of the figure. There may also be a horizontal band of compounds with $n_C \simeq 10$ also appearing with a low apparent volatility (high desorption temperature), ending in the prominent C_{10} outlier. This is consistent with thermal decomposition of dimers during the temperature programmed desorption itself.

The wide dynamic range of signals is compressed by the logarithmic symbol sizing, so in Fig. S2b we show the same data but with symbols shrinking to minimum size at a signal fraction of 0.01. This reveals that most of the particle signal is in 6 peaks. These were not used to form the empirical peak desorption volatility, and so it is encouraging that they fall near the 1:1 diagonal. The one prominent C_{10} outlier is barely visible to the left of the color scale.

S1.1 properties of volatility bins

While the peaks in Fig. 15 fall in color order with more volatile peaks generally appearing toward the upper left, the overall range of this behavior is compressed. The dVBS should reveal a set of compounds falling along a tight diagonal condensation line, all with $c^\circ < c^{xs}$, and a distribution of more volatile compounds falling well away from that line in decadal bins. Here we see a general diagonal, and general color order, but it is both more spread and more condensed than the simple model calculations suggest. The lobe of low-volatility species may well define the condensation line, but simply with experimental error consistent

with low signal to noise on each axis. However, based on the roughly 20 nm h^{-1} growth rate, we do expect compounds with $\log c^\circ \lesssim -2$ (purple, gray and ruddy in the color scale) to group together near the diagonal and the more volatile compounds (green and blue) to fall to the upper left. This is what we observe (the purple symbols are plotted last and so appear most prominently in the low volatility group, albeit with very low mass fractions).

To quantify the dVBS behavior we selected all peaks within each volatility bin (each separated by 1 order of magnitude) and determined the right-hand intercept of a line passing through the group. In theory that intercept would correspond to the excess concentration for species along the condensation line, and would fall above (to the upper left of) the condensation line. Fig. S3 shows those intercepts vs volatility for each sampling period and subsequent TPD ramp. For the lowest volatility bins the raw intercepts evolve slowly with time, but here for clarity we normalized the intercepts so the average for $\log c^\circ \leq -5$ is 7.5×10^5 (arbitrary units). This reveals the strong association between $\log c^\circ$ and intercept, with a roughly constant intercept for $\log c^\circ \ll -1$ and a steady increase for $\log c^\circ \gtrsim -1$. The figure shows the theoretically expected behavior if the volatility dynamics of the dVBS completely described the observations. While there is qualitative correspondence between what we observe and expect, it is not quantitative. The inflection point is either a few decades off of expectations, or not as sharp as expected, and the slope of the "semi volatile" region is shallower than the +1 expectation (though it approaches +1 for the highest volatility bins).

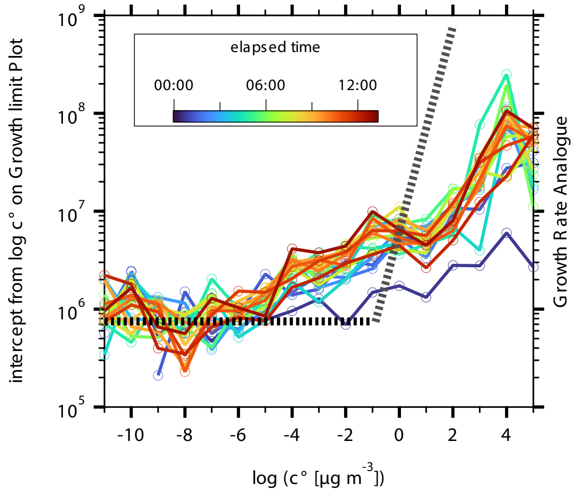

Fig. S3 Intercepts for each volatility bin during the entire run vs $\log c^\circ$ for each filter desorption, with (log-log) slopes constrained to 1.0. Intercepts rise slowly for $\log c^\circ \lesssim -1$ and rise sharply for $\log c^\circ \gtrsim 0$, with the overall curves rising over the course of the run, especially early in the run. The average of the intercepts for $\log c^\circ \lesssim -5$ for each filter desorption was normalized to the average of the final desorption and that factor was applied to the full range of volatility bins.

Fig. S3 includes all the temperature ramps during a run in which RH rose from 20% to 60%.[3] Inhibited uptake of SVOC would shift them to the left in the dVBS, as shown in Fig. 9. That would in turn shift the (right-hand) intercepts of these bins upward. At least for this run, there is no such behavior evident in Fig. S3. There is also no obvious sign of inhibited uptake of semi-volatile species associated with low relative humidity and glassy particles early in the run; that would cause semi-volatile species to shift notably to the left in Fig. 15) and so raise their (right-hand) intercept in Fig. S3. If anything the reverse is true, with the early intercepts falling below those later in the run (with higher RH), but the overall shape of the curves shows no systematic change during the run.

Notes and references

1 Q. Ye, M. Wang, V. Hofbauer, D. Stolzenburg, D. Chen, M. Schervish, A. Vogel, R. L. Mauldin III, R. Baalbaki, S. Brilke, L. Dada, A. Dias, J. Duplissy, I. E. Haddad, H. Finkenzeller, L. Fischer, X. He, C. Kim, A. Kurten, H. Lamkaddam, C. P. Lee, K. Lehtipalo, M. Leiminger, H. E. Manninen, R. Marten, B. Mentler, E. Partoll, T. Petäjä, M. Rissanen, S. Schobesberger, S. Schuchmann, M. Simon, Y. J. Tham, M. Vazquez-Pufleau, A. C. Wagner, Y. Wang, Y. Wu, M. Xiao, U. Baltensperger, J. Curtius, R. Flagan, J. Kirkby, M. Kulmala, R. Volkamer, P. M. Winkler, D. Worsnop and N. M. Donahue, *Environmental Science and Technology*, 2019, **53**, 12357–12365.

2 M. Wang, D. Chen, M. Xiao, Q. Ye, D. Stolzenburg, V. Hofbauer, P. Ye, A. L. Vogel, R. L. Mauldin III, A. Amorim, A. Baccarini, B. Baumgartner, S. Brilke, L. Dada, A. Dias, J. Duplissy, H. Finkenzeller, O. Garmash, X. He, C. R. Hoyle, C. Kim, A. Kvashnin, K. Lehtipalo, F. Lukas, U. Molteni, T. Petäjä, V. Pospisilova, L. L. J. Quéléver, M. Rissanen, M. Simon, C. Tauber, A. Tomé, A. C. Wagner, L. Weitz, R. Volkamer, P. M. Winkler, J. Kirkby, D. R. Worsnop, U. Baltensperger, J. Dommen, I. El Haddad and N. M. Donahue, *Environmental Science & Technology*, 2020, **54**, 7911–7921.

3 M. Surdu, H. Lamkaddam, D. S. Wang, D. M. Bell, M. Xiao, C. P. Lee, D. Li, L. Caudillo, G. Marie, W. Scholz, M. Wang, B. Lopez, A. A. Piedehierro, F. Ataei, R. Baalbaki, B. Bertozzi, P. Bogert, Z. Brasseur, L. Dada, J. Duplissy, H. Finkenzeller, X.-C. He, K. Höhler, K. Korhonen, J. E. Krechmer, K. Lehtipalo, N. G. Mahfouz, H. E. Manninen, R. Marten, D. Massabò, R. Mauldin, T. Petäjä, J. Pfeifer, M. Philippov, B. Rörup, M. Simon, J. Shen, N. S. Umo, F. Vogel, S. K. Weber, M. Zauner-Wieczorek, R. Volkamer, H. Saathoff, O. Möhler, J. Kirkby, M. Kulmala, F. Stratmann, A. Hansel, J. Curtius, A. Welti, M. Riva, N. M. Donahue, U. Baltensperger and I. E. Haddad, *Environmental Science & Technology*, 2023, **57**, 2297–2309.
