# Supplementary figures and images for: A diagonal volatility basis set to assess the condensation of organic vapors onto particles
Source: Environ Sci Atmos. 2025 Jul 21;5(9):1035–61. doi: 10.1039/d5ea00062a (PMC12314873; doi:10.1039/d5ea00062a)

intercept from  $\log c^\circ$  on Growth limit Plot

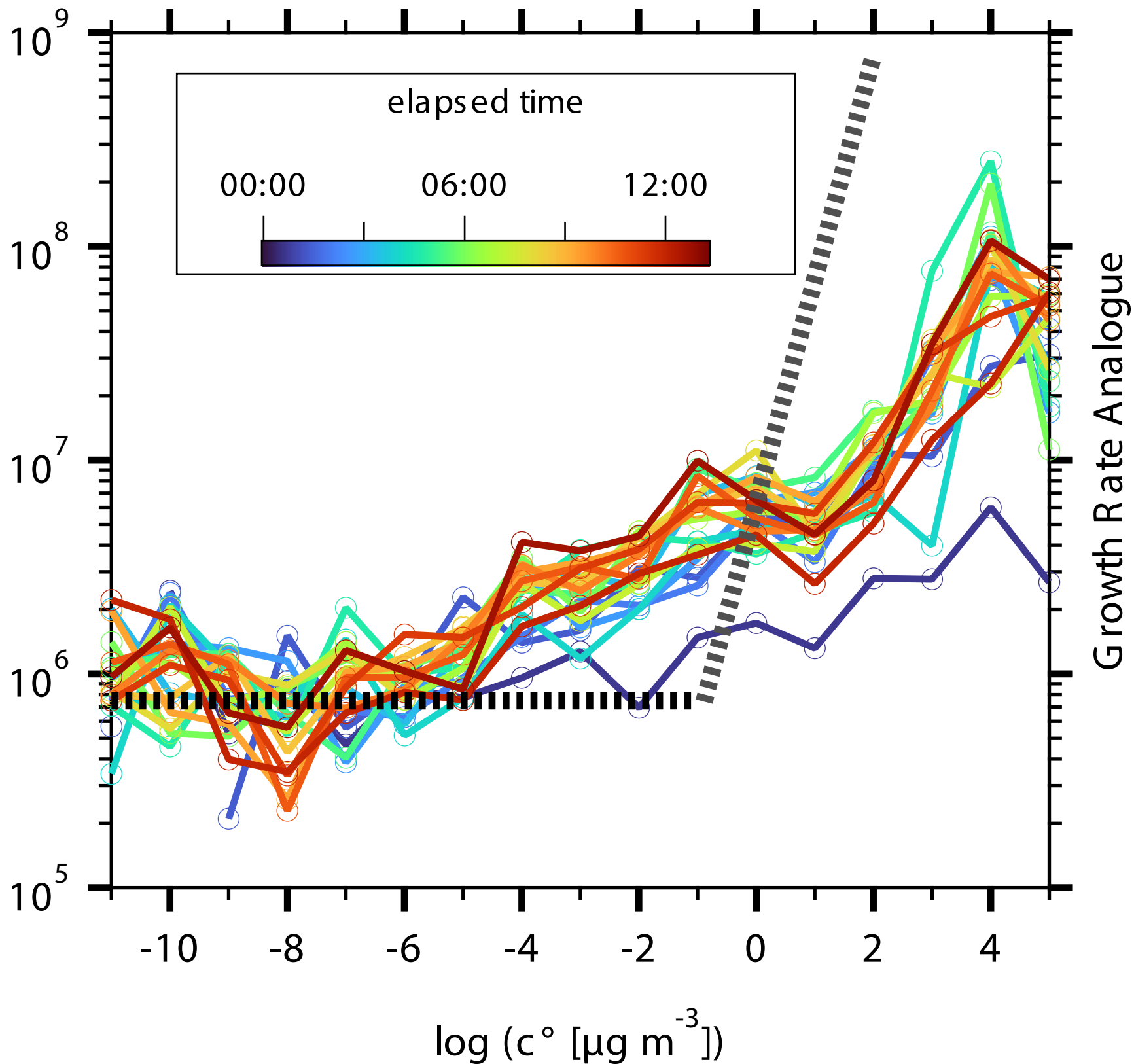

Supplement: EA-005-D5EA00062A-s002 [file EA-005-D5EA00062A-s002.zip › fig_s3.pdf]

$\log c^\circ$  (molecular composition)

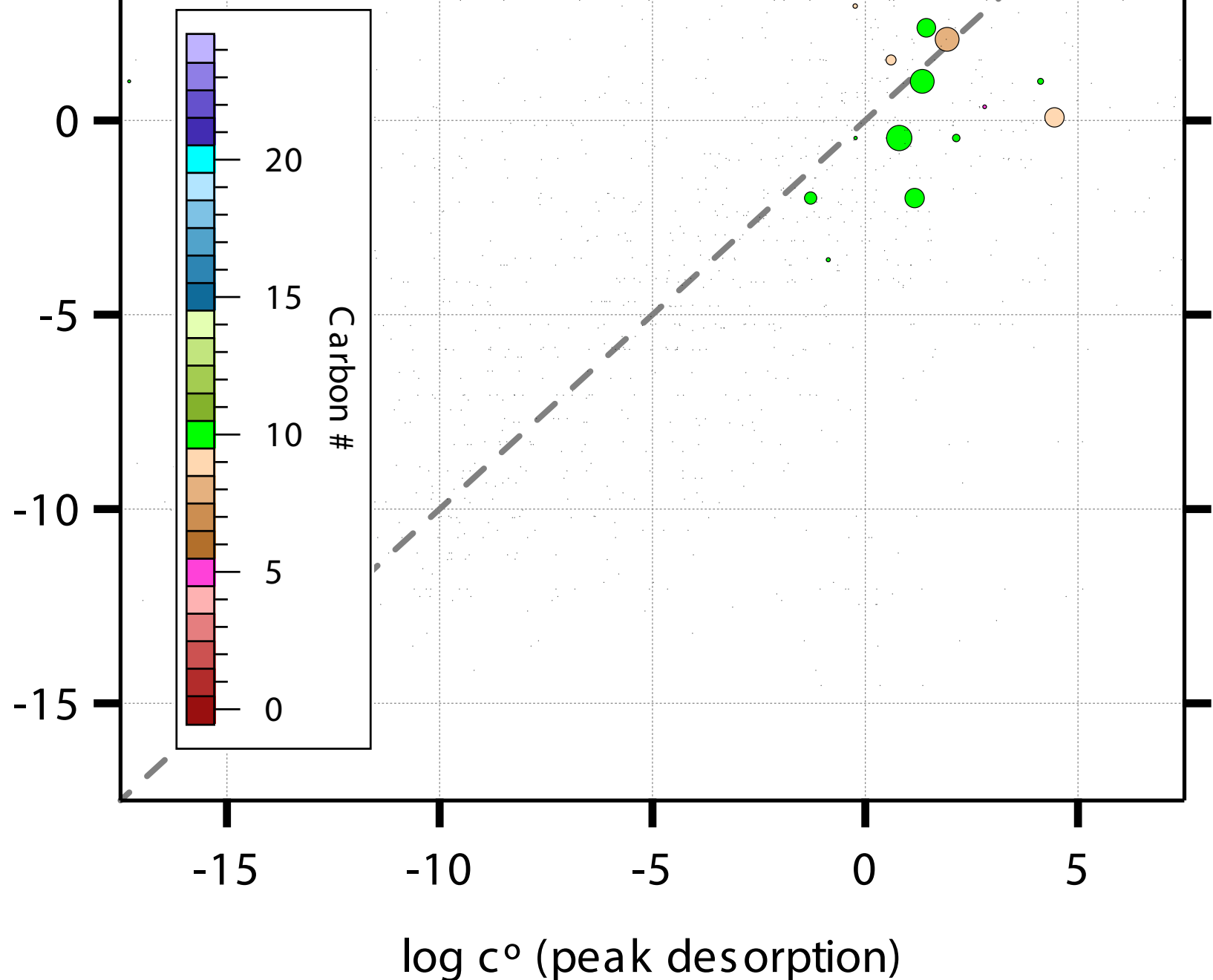

Supplement: EA-005-D5EA00062A-s002 [file EA-005-D5EA00062A-s002.zip › fig_s2b.pdf]

$\log c^\circ$  (molecular composition)

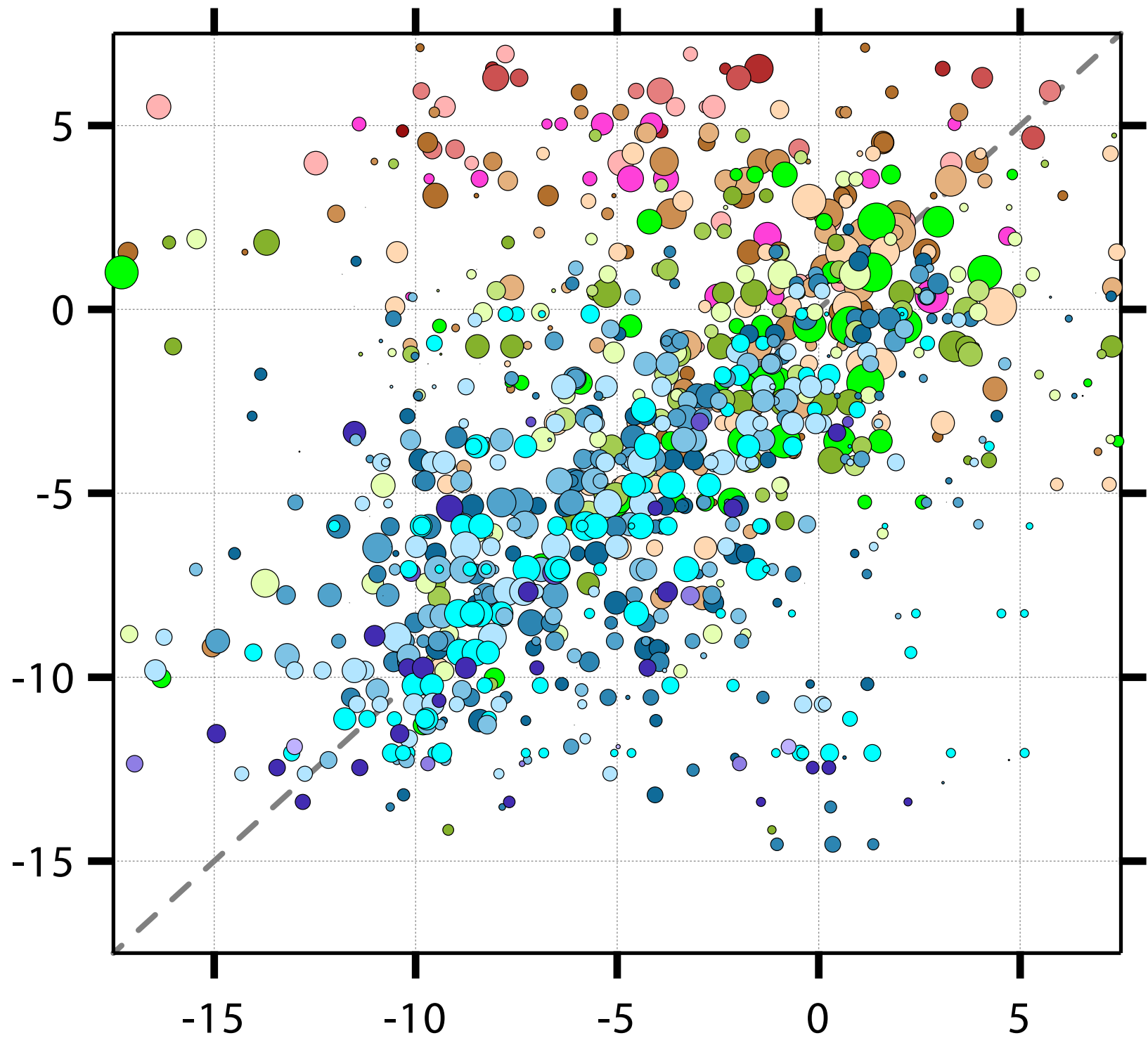

$\log c^\circ$  (peak desorption)

Supplement: EA-005-D5EA00062A-s002 [file EA-005-D5EA00062A-s002.zip › fig_s2a.pdf]

$\log c^\circ$  (molecular composition)

5  
0  
-5  
-10  
-15

60

80

100

120

$T_{\max}$  ( $^\circ\text{C}$ )

Carbon #

20

15

10

5

0

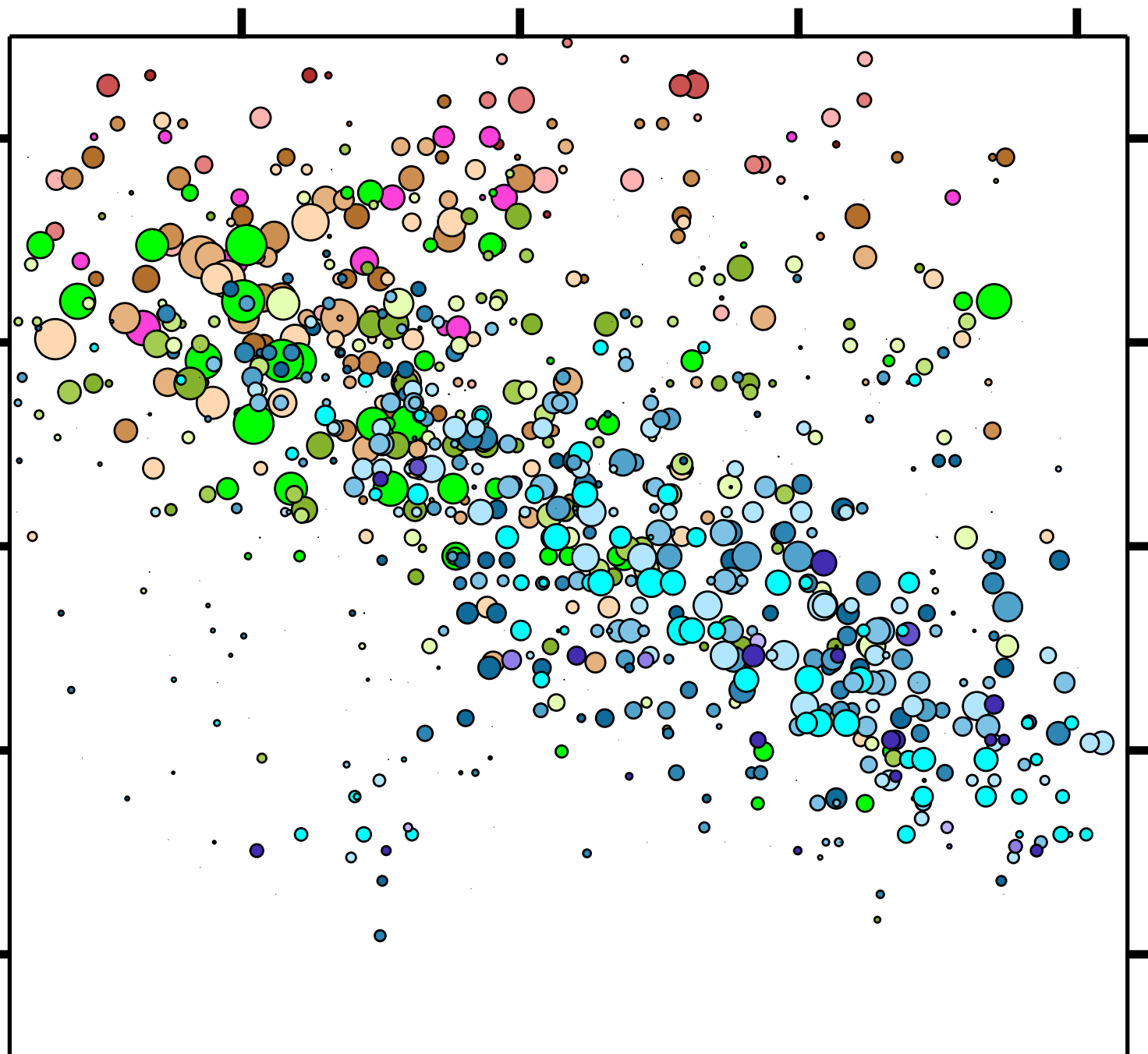

Supplement: EA-005-D5EA00062A-s002 [file EA-005-D5EA00062A-s002.zip › fig_s1.pdf]
